# In Silico Characterization of Pathogenic *ESR2* Coding and UTR Variants as Oncogenic Potential Biomarkers in Hormone-Dependent Cancers

**DOI:** 10.3390/genes16101144

**Published:** 2025-09-26

**Authors:** Hakeemah Al-Nakhle, Zainab Almoerifi, Layan Alharbi, Mashael Alayoubi, Rawan Alharbi

**Affiliations:** 1 Department of Clinical Laboratory Sciences, College of Applied Medical Sciences, Taibah University, Madinah 42353, Saudi Arabia; 2 Health and Life Research Center, Taibah University, Madinah 42353, Saudi Arabia

**Keywords:** *ESR2*, estrogen receptor beta (ERβ1), nsSNPs, UTR variants, protein stability, in silico analysis

## Abstract

**Background**: The *ESR2* gene encodes Estrogen Receptor-β1 (ERβ1), a putative tumor suppressor in hormone-dependent malignancies. Although ERβ biology has been studied extensively at the expression level, the functional impact of nonsynonymous SNPs (nsSNPs) and untranslated-region (UTR) variants in *ESR2* remains underexplored. **Methods**: We retrieved variants from Ensembl and performed an integrative in silico assessment using PredictSNP, I-Mutant, MUpro, HOPE, MutPred2, and CScape for pathogenicity, oncogenicity and structural stability; STRING/KEGG/GO for pathway context; RegulomeDB and polymiRTS for regulatory effects; and cBioPortal for pan-cancer clinical outcomes (breast (BRCA), endometrial (UCEC), and ovarian (OV)). We evaluated effects of nsSNPs on ERβ1 stability, ligand-binding/DNA-binding domains, co-factor recruitment, and post-transcriptional regulation. **Results**: Across tools, 93 missense nsSNPs were consistently predicted to be deleterious. Notably, several variants were found to destabilize ERβ1, particularly within the ligand-binding domains (LBD) and DNA-binding domains (DBD). Putative oncogenic drivers R198P and D154N showed high CScape scores and very low population frequencies, consistent with pathogenicity. Several substitutions were predicted to impair coactivator binding and disrupt interactions with key transcriptional partners, including *JUN*, *NCOA1*, and *SP1*. At the post-transcriptional level, rs139004885 was predicted to disrupt miRNA binding, while 3′UTR rs4986938 showed strong regulatory potential and comparatively high population frequency; by contrast, most other identified SNPs were rare. Clinically, pan-cancer survival analyses indicated worse overall survival (OS) in BRCA for *ESR2*-Altered cases (HR ≈ 2.25; q < 0.001), but better OS in UCEC (HR ≈ 0.24; q ≈ 0.014) and OV (HR ≈ 0.29; q < 0.001), highlighting a tumor-type-specific association. **Conclusions**: This integrative analysis prioritizes high-impact *ESR2* variants that likely impair ERβ1 structure and shows context-dependent clinical effects. Despite their generally low frequency (except for rs4986938), prospective validation linking variant class to ERβ expression and survival outcomes is needed to support biomarker development and therapeutic applications.

## 1. Introduction

The *ESR2* gene encodes Estrogen receptor beta 1 (ERβ1), a member of the nuclear hormone receptor superfamily that acts as a ligand-activated transcription factor [1,2,3,4].ERβ1 is associated with estrogen activity in tissues such as the prostate, brain, ovary, breast, and colon [1,2,3,4]. Unlike the growth-promoting ERα, ERβ1 mainly displays pro-apoptotic and anti-proliferative effects, making it a potential candidate for tumor suppression [5].

There are five structural domains of ERβ1, including the AF-1-containing N-terminal A/B domain, the DNA-binding domain (DBD), the hinge region, the ligand-binding domain (LBD) that contains AF-2, and the C-terminal F domain. The AF-1 region of ERβ1 is relatively shorter and has a lesser role in transcriptional activity compared to ERα [6]. However, DBD is essential for the specific recognition of estrogen response elements (EREs) in various target genes [7]. LBD is vital for ligand binding, transcriptional activation, and the recruitment of coactivators [8]. Structural differences in helix 12 among ERβ isoforms (ERβ5, ERβ4, and ERβ2) specifically affect their transcriptional capacity, with only ERβ1 showing full transcriptional activation [8]. Additionally, post-translational modifications such as O-GlcNAcylation, phosphorylation, and dimerization with ERα or itself influence ERβ function [9].

Mutations or genetic variations in ERβ1 domains, especially within the LBD and DBD, can significantly impair the receptor’s functional integrity. For instance, changes in the LBD helix 12 disrupt ligand binding and coactivator recruitment, thereby impacting downstream gene regulation [8,10]. Splice variants such as ERβ5, ERβ4, and ERβ2, which lack a functional helix 12, cannot transactivate DNA; however, they may influence ERβ1 function through dimerization [8]. Mutations in the zinc finger motifs of the DBD can impair DNA-binding ability and ER interaction with Stat5b, which is crucial for transcriptional synergy. These mutations alter how the receptor responds to ligands and antagonists—including tamoxifen and fulvestrant—and can cause conformational changes that disrupt ERβ-dependent signal transduction [11]. Genetic variations in the *ESR2* gene, especially nsSNPs, can cause dysfunctional ERβ1 by changing protein structure, receptor-cofactor and receptor–ligand interactions, and post-translational modifications. These changes disrupt ERβ1’s control of apoptosis, the cell cycle, and genes involved in differentiation. For example, polymorphisms like rs4986938 and rs1256049 have been linked to higher cancer risk across diverse populations [12,13,14,15].

nsSNPs—including those in untranslated regions (UTRs), introns, and promoter elements—can influence *ESR2* transcriptional activity, translational efficiency, and mRNA/protein stability. For instance, the TATA-box variant rs35036378 decreases promoter activity by about 50%, highlighting the functional impact of regulatory mutations. Similarly, polymorphisms within 3′-UTR microRNA recognition elements can interfere with seed pairing, reduce miRNA binding, and thus modify ERβ1 expression. Although these mechanisms are well understood for *ESR1*, evidence for miRNA-mediated regulation of *ESR2* remains relatively limited but is rapidly growing [14,16,17,18,19].

Polymorphisms in miRNA target sites within the 3′ UTRs of genes influence their binding affinity and may contribute to disease susceptibility. Conversely, functional variants of miRNA target sites in several genes, including the *ESR1* gene, have been identified, where the rs2747648 SNP modifies miR-453 binding, thereby affecting breast cancer risk [20]. Additionally, SNPs located within the 3′ UTRs of other genes are known to either enhance or impair miRNA interactions, subsequently impacting gene regulation [21].

The context-dependent nature of *ESR2* variants has been corroborated through their various associations with different cancers. Downregulation of ERβ1 is correlated with an unfavorable prognosis in breast cancer. The ERβ1 rs4986938 polymorphism is recognized to increase the risk of colorectal cancer, especially when high estrogen levels are present [22]. Similar correlations have been documented in lung and ovarian cancers [23], underscoring the role of tissue-specific factors and gene–environment interactions in the pathogenicity of *ESR2* variants [24].

Across hormone-related cancers, *ESR2* (ERβ1) acts as a context-dependent prognostic marker rather than a consistent one. In breast cancer, meta-analyses and large studies generally link higher ERβ/ERβ1 expression—particularly nuclear ERβ1—with better disease-free (DF), and OS, including among tamoxifen- or chemotherapy-treated groups; however, results differ depending on the isoform, analytical platform, and antibody specificity, and some reports show no association, highlighting the variability in assays and biology [25,26,27,28,29]. In ovarian cancer, pooled evidence indicates that ERβ’s favorable prognostic association emerges most clearly when ERβ1-specific clones (PPG5/10 or EMR02) are used, and single-cohort studies have linked cytoplasmic ERβ positivity to longer survival, highlighting subcellular localization and reagent effects [30,31]. In endometrial cancer, hormone-receptor expression overall tends to track with better outcomes, but ERβ-specific prognostic effects are less consistent across studies [32]. Pan-cancer analyses using TCGA further show that high *ESR2* mRNA can be favorable in several tumor types yet neutral or adverse in others, reinforcing tissue-, isoform-, and compartment-specific biology [33].

Polymorphisms in *ESR1* and *ESR2* have been consistently linked to susceptibility to polycystic ovary syndrome (PCOS) across various populations. In Chinese and Pakistani case–control groups, *ESR1* variants rs1999805 and rs9340799, respectively, were associated with higher PCOS risk [15,34]. A Tunisian cohort showed strong associations between PCOS and *ESR1* SNPs rs3798577 and rs2234693, as well as the *ESR2* SNP rs1256049 [35]. Consistent results were seen in women from Punjab, Pakistan, where *ESR1* variants rs2234693, rs8179176, rs9340799, and the *ESR2* variant rs4986938 were significantly associated with PCOS [15]. Overall, these findings suggest that genetic differences in the estrogen receptor contribute to PCOS risk across multiple ethnicities.

*ESR1* SNPs rs1554259481, rs755667747, and rs104893956, and seven *ESR2* SNPs, including rs140630557 and rs1463893698, are predicted to induce significant alterations in protein physicochemical properties and conformation. These structural modifications may impact receptor functionality, particularly regarding estrogen (E2) binding, which is essential for downstream hormonal signaling. Docking studies have indicated that SNPs rs1467954450 (*ESR1*), rs140630557, and rs1463893698 (*ESR2*) significantly reduce E2 binding affinity, potentially leading to hormonal imbalance and estrogen insensitivity in patients with PCOS [36].

The differentiation between ‘driver’ mutations, which facilitate tumor progression, and “passenger” mutations, which accrue without functional consequence, constitutes a considerable challenge within the domain of cancer genomics. It is imperative to enhance understanding of how non-coding variants and nsSNPs impact *ESR2* gene regulation and protein function to enable their accurate classification and evaluate their biological importance [37,38,39].

Despite increasing evidence that ERβ1 acts as a tumor suppressor in hormone-dependent cancers, comprehensive investigation of *ESR2* nsSNPs—especially within the LBD, DBD, and AF-1/AF-2 domains—remains limited, hindering the clinical application of variant profiling. Here, we perform an integrated in silico analysis that identifies and prioritizes harmful nsSNPs, assesses evolutionary conservation, and predicts their effects on ERβ1’s structure, physicochemical properties, and thermodynamic stability through molecular modeling. We map how variant-induced conformational changes affect domain architecture and combine these predictions with pan-cancer datasets to explore associations with tumor types and clinical outcomes, while distinguishing potential driver mutations from likely passenger mutations. Overall, this work clarifies the functional landscape of *ESR2* nsSNPs and promotes their potential as mechanistic biomarkers and targets for personalized endocrine therapy.

## 2. Materials and Methods

### 2.1. Data Retrieval and Allele-Frequency Aggregation and Population Variability

**Figure 1:**
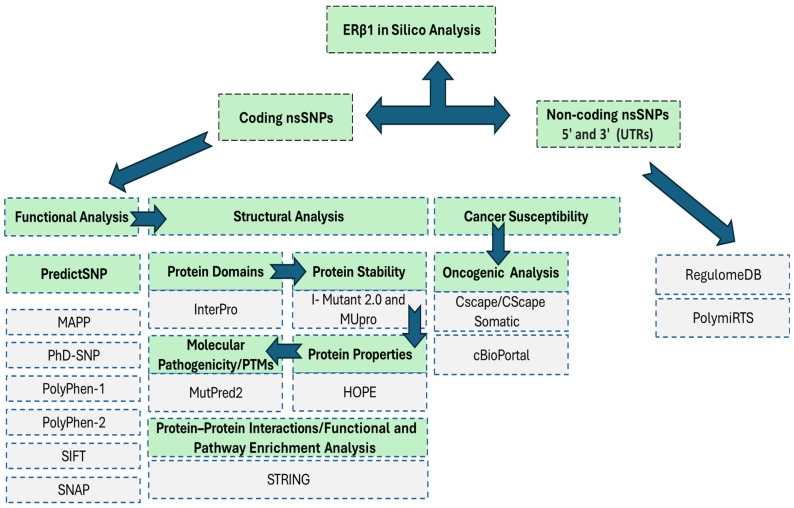
In Silico Analysis Pipeline for ERβ1 Coding and Non-Coding nsSNPs. The pipeline includes three main components: Functional Analysis, Structural Analysis, and Cancer Susceptibility Assessment for coding nsSNPs, while Regulatory Impact Assessment was used for non-coding variants (5′ and 3′ UTRs).

The *ESR2* gene, which encodes the ERβ1 protein, and its associated nsSNPs dataset (Ensembl Gene ID: ENSG00000140009) were retrieved from the ENSEMBL database [40]. Missense nsSNPs of the *ESR2* gene were identified using a missense variant filter. Figure 1 illustrates the overall workflow methodology. The corresponding ERβ1 protein sequence (FASTA format) was obtained from the UniProt database (UniProt ID: Q92731).

Allele frequencies (AF) for *ESR2* coding and 3′UTR variants were aggregated from gnomAD (GRCh38) and cross-referenced in Ensembl to confirm rsID and genomic positions. 

### 2.2. Functionally Damaging nsSNPs Identification

PredictSNP helped evaluate the potential functional impact of identified nsSNPs and determined whether each variant is harmful or not [41] (accessed on 22 January 2024). PredictSNP combines the results from several well-known algorithms, including SNAP, PolyPhen-1 and 2, MAPP, SIFT, and PhD-SNP, providing a comprehensive consensus prediction. This tool improves accuracy by using the different features of these algorithms, offering a strong estimate of the nsSNPs’ possible pathogenicity compared to single-method approaches.

### 2.3. nsSNPs Identification Within Conserved ERβ1 Protein Domains

The InterPro tool [42,43] (https://www.ebi.ac.uk/interpro/; accessed on 1 February 2024) mapped the identified nsSNPs to conserved ERβ1 protein domains. InterPro combines several protein signature databases—such as Pfam, PANTHER, PROSITE, Gene3D, PRINTS, SUPERFAMILY, ProDom, PIRSF, SMART, and TIGRFAMs—for detailed annotation of protein motifs and domains. This comprehensive approach helps identify conserved regions and evaluate the potential functional impacts of the nsSNPs. The ERβ1 protein sequence in FASTA format was obtained directly from InterPro.

### 2.4. Prediction of Protein Stability Alterations

Two predictive computational tools, MUpro 1.0 and I-Mutant 2.0, were used to evaluate how amino acid substitutions affect the stability of the ERβ1 protein. I-Mutant 2.0 (https://folding.biofold.org/i-mutant/i-mutant2.0.html; accessed on 8 February 2024) is an algorithm based on Support Vector Machines (SVM) that predicts changes in protein stability caused by single-site mutations. It categorizes mutations as either destabilizing or stabilizing with roughly 80% accuracy, especially when the three-dimensional structure of the protein is available. Missense nsSNPs were analyzed under standard conditions (25xc C, pH 7.0) throughout this study. The software predicted the change in Gibbs free energy (ΔΔG), offering a qualitative assessment of protein stability. Additionally, it provided a Reliability Index (RI) for each prediction [44].

MUpro 1.0 (http://mupro.proteomics.ics.uci.edu/; accessed 8 February 2024) was used alongside other methods to assess the effects of mutations on protein stability. MUpro 1.0 combines SVM and neural networks, trained extensively on mutation datasets, and achieves over 84% accuracy through 20-fold cross-validation. Structural information is not required for MUpro, which works solely on sequence data. It outputs a predicted ΔΔG value and a confidence score ranging from −1 to 1. Scores below 0 indicate decreased stability, while scores above 0 suggest increased stability [45]. All predictions were conducted using default settings and interpretations, following established analysis criteria.

### 2.5. nsSNPs Structural Impact Assessment on Human ERβ1 Protein

The potential structural impacts of nsSNPs on the human ERβ1 protein were evaluated using the Project HOPE platform (Have Our Protein Explained) version 1.1.1 (https://www3.cmbi.umcn.nl/hope/; accessed on 8 February 2024). This tool requires the input of protein sequences (FASTA format) along with detailed SNP information. It consolidates data from the Distributed Annotation System (DAS), WHAT IF modeling software, and the UniProt database to provide comprehensive insights into structural alterations.

The Project HOPE platform builds a homology-based model to support a thorough evaluation of mutation effects. This tool explains the functional and structural impacts of each nsSNP through comparative analysis of mutant and wild-type proteins. The output includes detailed annotations, visual diagrams, and interactive animations.

### 2.6. nsSNPs Molecular Pathogenicity Evaluation

The MutPred2 algorithm was utilized (http://mutpred.mutdb.org; accessed on 8 February 2024) to analyze the molecular pathogenicity and prospective functional implications of amino acid substitutions within the ERβ1 protein. MutPred2 assesses the pathogenic potential of nsSNPs by estimating the likelihood of deleterious amino acid substitutions at a significance threshold of *p* < 0.05 [46].

It integrates various functional and structural properties of proteins, including transmembrane regions, secondary structures, signal peptides, binding affinities (metal and macromolecular), catalytic activity, allosteric regulation, and post-translational modifications (PTMs). Variations in amino acid residues may disrupt these characteristics, potentially leading to alterations in protein stability, loss or gain of PTM sites, compromised structural integrity, and impaired interactions with other biomolecules. These phenomena ultimately influence protein function and behavior at the molecular level.

The human ERβ1 protein sequence (FASTA) and detailed amino acid substitutions were submitted to the MutPred2 server. The algorithm produced a probabilistic score indicating the potential association of each nsSNP with deleterious or disease-related effects. Additionally, it provided a list of predicted molecular alterations accompanied by corresponding *p*-values. The results delineated the specific functional disruptions associated with each variant within a designated statistical threshold.

### 2.7. Oncogenic and Phenotypic Characterization

CScape and CScape Somatic tools contributed to the assessment of oncogenic potential for the identified nsSNPs. CScape assigned predictive scores to each nsSNP and classified variants as benign or oncogenic with high confidence. These classifications were derived from machine learning models trained on extensive cancer genomics datasets. Prediction accuracy was approximately 92% for coding regions and 76% for non-coding regions [47]. The scoring system reflects the likelihood of a variant exhibiting oncogenic characteristics, with higher scores indicating a stronger tumorigenic potential. Consequently, CScape prioritized variants according to their predicted oncogenic potential for subsequent investigation. Additionally, CScape Somatic assisted in evaluating somatic point mutations identified within coding regions of the cancer genome. This tool distinguishes mutations as oncogenic, potential cancer drivers, or neutral, thereby aiding in mutation interpretation in the context of oncogenesis [48]. CScape-somatic was trained using pan-cancer somatic mutation data (COSMIC, TCGA) and incorporated data on protein structure, mutational hotspots, and evolutionary conservation. High-confidence driver mutations were indicated by scores > 0.7.

### 2.8. Patient Survival and Pan-Cohort Clinical Outcome Analysis

We analyzed cBioPortal aggregated studies for three tumor types—breast carcinoma (BRCA), uterine corpus endometrial carcinoma (UCEC), and ovarian cancer (OV)—and extracted survival data stratified by *ESR2* alteration status. Samples were classified as Altered if they contained any reported *ESR2* mutation or nsSNP, and Unaltered otherwise. For each tumor type, Kaplan–Meier estimates were compared using the log-rank test; when available from the cBioPortal export, false-discovery rate (FDR)-adjusted q-values were recorded. Cox proportional hazard models provided hazard ratios (HRs) with 95% confidence intervals (CIs); pairwise HR matrices interpret the hazard of the column group relative to the row group. Time is measured in months, and medians are reported as “NA” when not reached. Endpoints included OS, and when available, progression-free survival (PFS), disease-specific survival (DSS), and disease-free or relapse-free survival (DFS/RFS). No multivariable adjustment was made. Analyses were conducted on exports accessed on 13 September 2025.

### 2.9. Prediction of Protein–Protein Interactions

The Search Tool for the Retrieval of Interacting Genes/Proteins (STRING) database (http://string-db.org; accessed on 25 February 2024) was employed to elucidate *ESR2* protein–protein interactions (PPIs) [49,50]. STRING characterizes proteins’ functional associations utilizing various sources, including computational prediction, literature, and experimental data.

The STRING database contains 24,584,628 proteins from 5090 species, enabling comprehensive interactome analyses. The human *ESR2* protein sequence (FASTA) was submitted to the database, resulting in the identification of potential interaction partners. The findings outlined a detailed interaction network, annotated with confidence scores to evaluate the reliability of each predicted interaction. These insights into the interaction data offer a deeper understanding of the functional role of *ESR2* within molecular and cellular pathways.

### 2.10. Functional and Pathway Enrichment Analysis

The functional analysis encompassed protein annotation and refinement within the network based on their specific roles. Pathway enrichment was primarily conducted using Gene Ontology (GO) terms, which included cellular components, biological processes, and molecular functions, supplemented by pathway-based analyses. Such functional analysis is essential for understanding the biological and physical significance of the network. The STRING database was utilized to perform GO and KEGG pathway enrichment analyses.

### 2.11. Analysis of the Functional Significance of Non-Coding SNPs (ncSNPs) in the ESR2 Gene: A RegulomeDB-Based Evaluation of Regulatory Function

ncSNPs were identified from the non-coding regions of the ESR2 gene using the genomeAD and ENSEMBL databases. RegulomeDB v2 was used to map ncSNPs to human genome regulatory elements [51]. SNP identifiers were annotated with a minor allele frequency (MAF, <0.001) from the 5′ or 3′ UTRs, obtained from the Ensembl database. The results included information about chromosomal position, dbSNP ID, score, and rank. Identifying functional variants in these regions is important because they may affect regulatory functions. RegulomeDB v2 helps predict and rank ncSNPs as regulatory elements by integrating data from ENCODE ChIP-seq, DNase I hypersensitive sites, FAIRE, dsQTLs, and eQTLs. The system categorizes ncSNPs into six groups based on their potential effects on gene expression or transcription factor binding (Table S2).

### 2.12. Analysis of 3UTR SNPs Influence on miRNA Binding Sites

The PolymiRTS Database 3.0 was utilized to analyze UTR variants (both 3′ and 5′) (https://compbio.uthsc.edu/miRSNP/; accessed on 22 November 2024), and their effects on miRNA binding sites were assessed [52]. A specific variant ID was input into the tool, which subsequently generated the corresponding miRNA ID, Context+ score, and functional annotation. Variants were classified into four functional categories: “D” (disruption of conserved miRNA-binding sites), “N” (disruption of non-conserved sites), “C” (creation of novel miRNA-binding sites), and “O” (absence of ancestral allele data). The Context + score provided a quantitative measure of impact, where more negative values indicated a higher potential for disease association due to disrupted miRNA targeting.

## 3. Results

### 3.1. Identification of nsSNPs’ Functional Impact Within the ESR2 Gene

Multiple in silico prediction tools were used to analyze 93 nsSNPs, revealing potential functional impacts of missense variants in the *ESR2* gene. All platforms consistently predicted the identified missense nsSNPs as either “Deleterious” by PredictSNP and combined SNAP, MAPP, SIFT, and PhD-SNP tools, or “Damaging” by PolyPhen-1 and PolyPhen-2. This agreement across various algorithms indicates a strong consensus that amino acid substitutions may negatively affect the protein’s structural and functional properties (Table 1). Notably, SNPs (rs766405281) with multiple amino acid substitutions at the C191 position—such as C191G, C191Y, C191R, and C191S—were consistently predicted as deleterious, suggesting these sites are likely critical structural or functional hotspots within the protein. Likewise, recurrent mutations at residues R388, R198, and L339 further emphasize regions of potential functional significance (Table 1).

### 3.2. Domain Structure of ESR2 (ERβ1) and Distribution of Oncogenic nsSNPs Using InterPro

**Figure 2:**
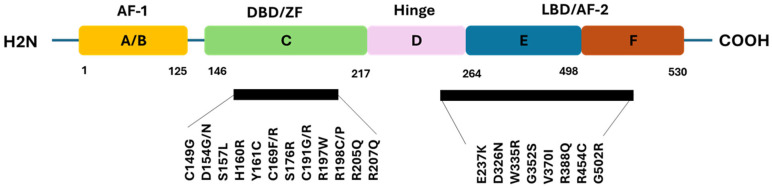
Schematic representation of the structural and functional domains of the ERβ1 protein with localization of predicted oncogenic driver nsSNPs. The receptor comprises six major domains (A–F): the A/B domain (yellow) containing the AF-1; the C domain (green) representing the DBD; the D domain (pink) corresponding to the hinge region harboring the nuclear localization signal (NLS); the E domain (blue) encoding the LBD and AF-2; and the F domain (orange), a variable C-terminal region with modulatory roles. In silico analysis identified oncogenic driver nsSNPs clustering in critical functional regions (highlighted in bold black).

InterPro annotation confirmed that the ERβ1 protein, which comprises six domains (A–F) (Figure 2). The A/B domain (AF-1; residues 1–125) for ligand-independent activation, the C domain (DBD; residues 146–217) mediates DNA binding to estrogen response elements (EREs), the D domain (hinge; residues 217–264) includes the nuclear localization signal (NLS), the E/F domain (LBD; residues 264–530) harbors AF-2 for ligand-dependent activation, dimerization, and co-regulator recruitment (Figure 2). 

Pathogenic variant mapping revealed several oncogenic driver nsSNPs predicted by CScape and CScape-somatic in the Section 3.7. For example, the oncogenic nsSNPs within the DBD such as C149G and D154G/N may impair ERE recognition. In the LBD, G352S is predicted to attenuate coactivator recruitment and destabilize estrogen signaling. These high-risk variants cluster in domains essential for ERβ1 transcriptional activity, suggesting a mechanistic link between *ESR2* alterations, impaired receptor function, and oncogenic signaling in hormone-dependent cancers.

### 3.3. Missense nsSNPs’ Predicted Effects on Protein Stability Across Functional Domains

I-Mutant analysis indicated that most variants reduced protein stability, with RI values ranging from 1 to 9. The analysis mostly showed negative ΔΔG values, suggesting destabilizing effects, especially in the critical LBD and DBD regions. Variants from C149G, D154G, and L380P exhibited strong destabilization, with significant negative ΔΔG values of −3.26, −2.25, and −3.44 kcal/mol, respectively.

MUpro also predicted negative ΔΔG values, indicating that most variants decrease protein stability. One tool suggested that a small subset of mutations including S112L, K208M, S529F, and T290I increased protein stability; however, their small ΔΔG values indicated a limited stabilizing effect.

LBD and DBD mutations were prevalent and consistently forecasted to destabilize *ESR2*, thereby indicating their potential functional importance. Notably, several residues such as L339, C191, and R198 exhibited multiple substitutions, which were predicted to affect protein stability adversely (Table 2).

### 3.4. Structural Implications of ESR2 nsSNPs on Protein Conformation

HOPE-based structural predictions indicated that nsSNPs can substantially affect *ESR2*’s molecular structure by inducing various changes in residue properties such as conservation patterns, size, hydrophobicity, and charge, as well as in evolutionary conserved regions.

Cysteine residues (C149G, C152Y, Y161C, C191Y/S/G/R, C169F/R, R227C, and R444C) were impacted by most of the analyzed variants, leading to the loss of aromatic interactions and crucial disulfide bonds. These substitutions often caused steric clashes due to larger side chains (C152Y, C191Y) or destabilized the protein core by inducing smaller residues like C191G. Notably, glycine and proline residues (G542V, G502R, G352S, G162R, and P156R) were disruptive, affecting backbone flexibility and secondary structure stability, which suggests potential folding defects (Table S1).

Charge-reversing mutations (K244E, E211K, and E237K) and loss-of-charge variants (D326N, D154N/G, D303N, R388Q, R329Q, R207W/Q, R205Q, R198P/C, and R197W/Q) significantly affected electrostatic interactions. These changes could impair DNA recognition, ligand binding, and protein dimerization, especially in conserved functional domains. Additionally, hydrophobic core stability was influenced by increased hydrophobicity (V370I, S157L, and N179Y) or the loss of hydrophobic packing (W535R), which may lead to protein aggregation or misfolding (Table S1).

Several high-impact variants were identified in highly conserved regions (G502R, D303N, R388Q, G352S, and V370I), highlighting their functional importance. Mutations involving glycine (G352S, G502R) and charged residues (E237K, R554S) in conserved regions are concerning, as they are crucial for maintaining structural integrity and regulating molecular interactions. Therefore, *ESR2* nsSNPs could alter receptor function through various structural mechanisms, requiring further experimental research to determine their phenotypic effects.

### 3.5. Predicted Pathogenicity and Molecular Impact of ESR2 nsSNPs

MuPred2, a predictive model, was used to analyze the potential molecular effects of selected nsSNPs in the *ESR2* gene. The model integrated protein-level features to evaluate mutation pathogenicity and related molecular mechanisms. MuPred2 scores (ranging from 0 to 1) and the associated functional changes—such as structural, biochemical, and post-translational modifications—were assessed for each variant, especially those with *p*-values < 0.05. All 29 mutations examined had MuPred2 scores above 0.6, indicating a high likelihood of pathogenicity. Notably, several variants scored over 0.9, including G162R (0.910), C169R (0.936), and C169 R/F (0.936/0.919). These findings strongly suggest deleterious effects. Changes in the interface and disordered regions, observed in numerous mutations like R198C, H160R, and C169R, were the most affected molecular mechanisms. These alterations could impact protein–protein interactions and post-translational modifications such as ubiquitylation (K300), disulfide linkages (C191G/R/S/Y), and GPI-anchor amidation (N189, N407), indicating significant effects on protein localization and regulation. Structural disturbances, including the loss or gain of helices or strands and modifications of metal-binding capacity—impact overall protein function and folding. Variants at C191 (C191Y, C191G, C191S, and C191R) consistently showed loss of disulfide bonds, disruption of DNA-binding and transmembrane regions, and alterations in glycosylation or GPI-anchor sites near residues like N189. Additionally, mutations at N189Y, R198P, and D154N exhibited multiple functional and structural impacts. Overall, the results highlight the importance of residues within DNA-binding and transmembrane regions as hotspots for functional mutations in ESR2 (Table 3).

### 3.6. Tumor-Type–Specific Prognostic Impact of ESR2 Alterations (Adverse in BRCA; Protective in UCEC/OV)

**Figure 3:**
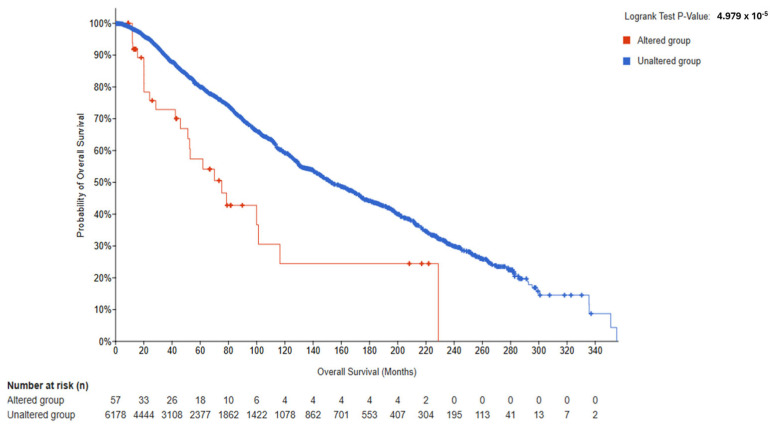
Kaplan–Meier overall survival by *ESR2* alteration status in BRCA. Tumors were classified as Altered (any reported *ESR2* mutation or nsSNP; *n* = 57) or Unaltered (*n* = 6178). Median OS: 75.23 months (Altered; 95% CI, 51.20–NA) vs. 152.93 months (Unaltered; 95% CI, 146.39–164.57). Log-rank *p* = 4.98 × 10^−5^; FDR-adjusted q = 1.25 × 10^−4^. Cox model: HR (Altered vs. Unaltered) = 2.251 (95% CI, 1.235–4.100). Tick marks indicate censoring; time in months; medians reported as “NA” when not reached. Tick marks indicate censoring, time in months.

In BRCA the OS-evaluable set (*N* = 6235; altered = 57; unaltered = 6178), survival was significantly worse in the altered group (log-rank *p* = 4.98 × 10^−5^; q = 1.25 × 10^−4^), with HR (altered vs. unaltered) = 2.251 (95% CI, 1.235–4.100) and medians of 75.23 versus 152.93 months. PFS and RFS also favored the unaltered group (PFS *p* = 4.52 × 10^−5^, q = 1.25 × 10^−4^; RFS *p* = 0.0195, q = 0.0244), whereas DFS was not significant (*p* = 0.156) (Figure 3, Table S3).

**Figure 4:**
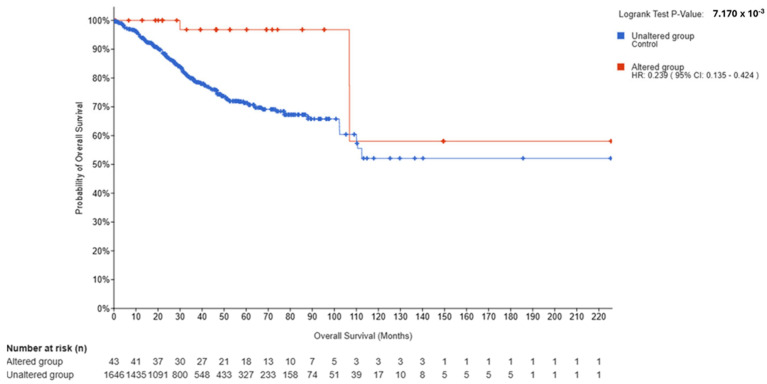
Kaplan–Meier overall survival by ESR2 alteration status in UCEC. Altered (n = 43; events = 3) vs. Unaltered (n = 1646; events = 328). Log-rank *p* = 7.170 × 10^−3^; FDR-adjusted q = 0.0143. HR (Altered vs. Unaltered) = 0.239 (95% CI, 0.135–0.424). Median OS not reached in the export (NA). Tick marks indicate censoring, time in months.

In UCEC (*N* = 1689; Altered = 43; Unaltered = 1646), OS was significantly better in the Altered group (log-rank *p* = 7.170 × 10^−3^; q = 0.0143), with HR (Altered vs. Unaltered) = 0.239 (95% CI, 0.135–0.424) and events 3/43 versus 328/1646. Progression-free survival (PFS) was also significant (*p* = 6.730 × 10^−3^; q = 0.0143), disease-specific survival (DSS) was nominally significant but borderline after FDR correction (*p* = 0.0399; q = 0.0532), and disease-free survival (DFS) was not significant (*p* = 0.0903; q = 0.0903) (Figure 4, Table S3).

**Figure 5:**
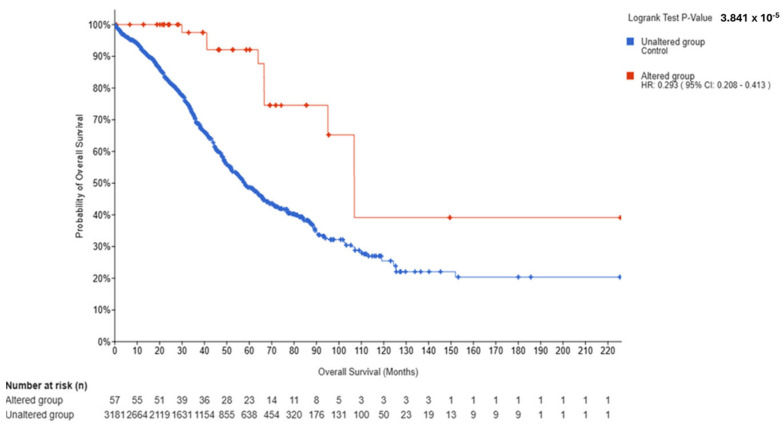
Kaplan–Meier overall survival by *ESR2* alteration status in OV. Altered (*n* = 57; events = 10) vs. Unaltered (*n* = 3181; events = 1245). Median OS: 106.88 months (Altered; 95% CI, 95.07–NA) vs. 58.05 months (Unaltered; 95% CI, 55.43-62.12). Log-rank *p* = 3.841 × 10^−5^; FDR-adjusted *q* = 1.537 × 10^−4^. HR (Altered vs. Unaltered) = 0.293 (95% CI, 0.208–0.413). Tick marks indicate censoring; time in months; medians reported as “NA” when not reached.

In OV (*N* = 3238; Altered = 57; Unaltered = 3181), OS was significantly better in the Altered group (log-rank *p* = 3.841 × 10^−5^; q = 1.537 × 10^−4^), with HR (Altered vs. Unaltered) = 0.293 (95% CI, 0.208–0.413), medians of 106.88 vs. 58.05 months, and events of 10/57 vs. 1245/3181. Additional endpoints were consistent: PFS (*p* = 4.471 × 10^−4^; q = 8.941 × 10^−4^), DFS (*p* = 2.577 × 10^−3^; q = 3.390 × 10^−3^), and DSS (*p* = 3.390 × 10^−3^; q = 3.390 × 10^−3^), all favored the Altered group (Figure 5, Table S3).

Cross-tumor synthesis. The direction of association diverged by tumor type: BRCA showed adverse outcomes for *ESR2*-Altered cases (HR > 1), whereas UCEC and OV showed favorable outcomes (HR < 1), each with FDR-significant OS separation. Endpoint patterns were partially consistent with the OS signal—strongest and most internally consistent in OV (all endpoints significant), mixed in BRCA (PFS/RFS significant; DFS not), and modest in UCEC (OS/PFS significant; DSS borderline; DFS not). These patterns occurred in the setting of marked sample-size asymmetry (Altered *N* ≈ 43–57 in each cohort) and low event counts in Altered groups (e.g., UCEC 3/43; OV 10/57), which influence the precision of HR estimates.

### 3.7. CScape-Somatic (v1.0) and CScape (v2.0) Tools-Based Oncogenicity Determination

The oncogenic potential of *ESR2* gene variants was assessed using the CScape and CScape-somatic prediction tools, which assign oncogenicity scores from 0 to 1 and classify mutations as either drivers or passengers based on their role in cancer development. Higher scores suggest a greater likelihood of being drivers. At the same time, allele frequency (AF) data from GenomeAD and tumor-type associations from CBioPortal helped evaluate the importance of these mutations. Many variants analyzed were identified as high-confidence oncogenic drivers, with CScape scores over 0.90 for multiple substitutions such as R198P (0.9744), C149G (0.9295), C191G (0.9656), D154G (0.9292), and D154N (0.9626). Supporting evidence from CScape-somatic predictions confirmed these results by finding several driver mutations, including R454C, R198C, and D154N. Notably, residue C191, which was frequently altered across various C191R/S variants with consistently high oncogenic scores, was predicted as a driver. These results suggest that this position is a potential mutational hotspot. This observation agrees with previous MuPred2 predictions—both structural and functional—further supporting its importance in disease development.

CBioPortal data revealed cancer-type-specific associations of several variants, where *R197W* was linked to papillary, stomach, and colon adenocarcinomas; *R207Q* to stomach adenocarcinoma; *D326N* to breast invasive ductal carcinoma; *D303N* to chromophobe renal cell carcinoma; *V370I* to renal clear cell carcinoma; *G352S* to uterine endometrioid carcinoma; *G502R* to rectal adenocarcinoma; and *R388Q* to lung squamous cell carcinoma. Most of these driver-classified variants exhibited significantly low allele frequencies in the general population (AF < 0.00001), which is consistent with their somatic origin and potential pathogenicity in tumorigenesis (Table 4).

### 3.8. Determination of ESR2 Protein Interactions

Analysis of ERβ1 PP interactions in STRING revealed a diverse network encompassing signaling mediators, structural proteins, and transcriptional co-regulators. A high-confidence interaction map (score ≥ 0.9) identified associations with nuclear receptor coactivators (*SRC, NCOA1-3, NCOA2,* and *NCOA3*) and the mediator complex subunit *MED1*, thereby supporting *ESR2*’s involvement in ligand-dependent transcription. A closely connected cluster of *NCOA1-3* and *MED1* indicated a coordinated increase in *ESR2*-driven gene expression.

**Figure 6:**
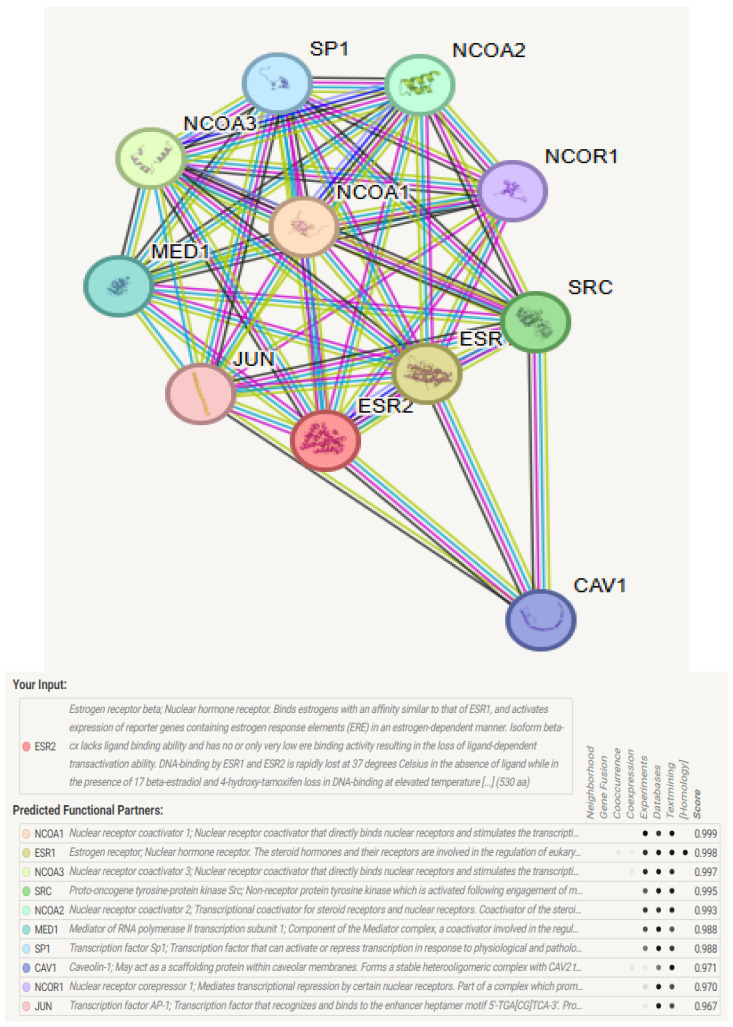
The Protein–Protein Interaction (PPI) network of *ESR2* based on the STRING database. It illustrates ESR2’s direct and indirect interactions with various proteins such as signaling mediators, transcriptional co-regulators, and nuclear receptor coactivators. Important partners include *NCOA1/2/3*, *MED1*, *SRC*, *NCOR1*, *SP1*, *CAV1*, and *JUN*, which enhance, connect, modulate, or support ESR2 activity and signaling.

*NCOR1*, a corepressor, facilitates *ESR2*’s dual regulatory roles within estrogenic tissues. Its interactions with *SP1* and *JUN* suggest a crosstalk between estrogen and stress pathways involved in gene regulation. *CAV1* indicates *ESR2*’s participation in membrane signaling beyond its genomic functions. ESR1 was identified as the primary heterodimerization partner, underscoring potential compensatory mechanisms in tissues co-expressing both receptors. *NCOA2* and *MED1* exhibited the highest binding affinities for wild-type *ESR2* (STRING scores: 0.99 and 0.98, respectively), whereas *CAV1* and *JUN* demonstrated context-dependent associations (scores: 0.97–0.96) (Figure 6).

### 3.9. Functional Enrichment Analysis of ESR2-Associated Pathways

GO biological processes and KEGG pathways derived from the STRING protein association networks were utilized for pathway enrichment analyses to elucidate the functional landscape of ESR2 and its associated protein network.

**Figure 7:**
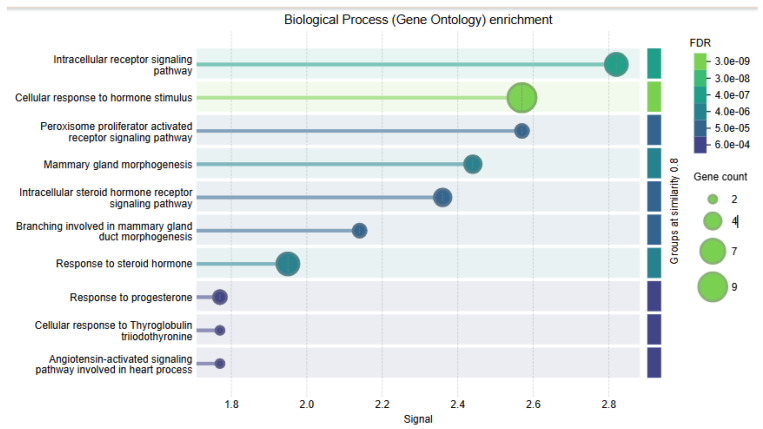
Gene Ontology (GO) enrichment analysis of biological processes associated with *ESR2*-linked genes. The bubble plot displays enriched biological processes, with the x-axis representing the enrichment signal. Bubble size indicates the number of enriched genes, while bubble color reflects the false discovery rate (FDR), ranging from 3.0 × 10^−9^ to 6.0 × 10^−4^. Groups were clustered at a similarity threshold of 0.8. Top enriched processes include intracellular receptor signaling, cellular response to hormone stimulus, steroid hormone receptor signaling, and mammary gland morphogenesis, underscoring the hormone-dependent regulatory functions of *ESR2*.

Gene Ontology (GO) enrichment analysis identified proteins interacting with *ESR2* that are associated with hormone development and signaling pathways. Notably, the primary enriched terms included “response to hormone stimulus”, “intracellular receptor signaling”, “mammary gland morphogenesis”, and “PPAR signaling”. These terms demonstrated high enrichment scores (ranging from 1.8 to 2.8) and extremely low false discovery rates (FDRs) of 3.0 × 10^−9^, underscoring their statistical significance. Several terms were linked to steroid hormones and mammary gland development, thereby elucidating the biological functions of ESR2. The size of the bubbles depicted in the figure corresponds to the gene count, with some terms involving up to nine genes, thereby emphasizing *ESR2*’s role in hormonal processes (Figure 7).

**Figure 8:**
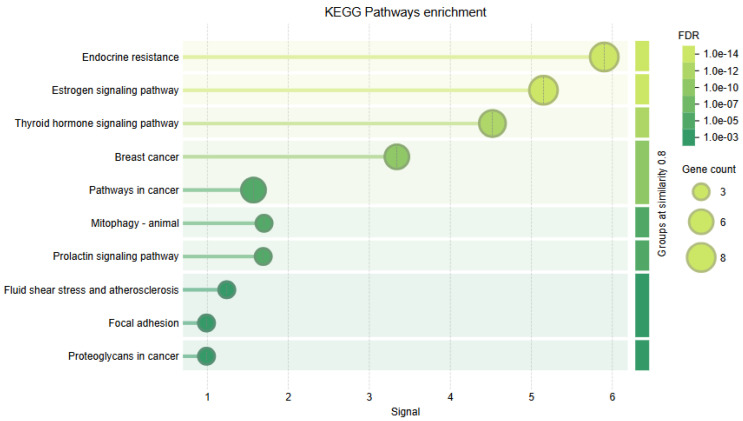
KEGG pathway enrichment analysis of ESR2-associated genes. The bubble plot displays significantly enriched pathways, with the x-axis representing the enrichment signal score. Bubble size corresponds to the number of enriched genes, while bubble color indicates the false discovery rate (FDR), with lighter shades reflecting higher significance. Notably enriched pathways include endocrine resistance, estrogen signaling, thyroid hormone signaling, breast cancer, and general cancer pathways, highlighting the oncogenic relevance of *ESR2* alterations.

Complementary KEGG pathway enrichment analysis further corroborated *ESR2*’s involvement in oncogenic pathways and hormonal signaling. Notably, estrogen signaling, endocrine resistance, breast cancer, and thyroid hormone signaling emerged as the primary pathways, exhibiting signal values up to 6.0 and lower false discovery rates (FDRs) of 1.0 × 10^−14^. These findings highlight *ESR2*’s regulatory functions in hormone-related resistance mechanisms and malignancies. The enrichment of “Prolactin signaling” and “fluid shear stress and atherosclerosis” suggests broader physiological significance (Figure 8).

These enrichment profiles robustly endorse *ESR2*’s essential function in the regulation of hormone response, development, and oncogenic pathways. Furthermore, they highlight their involvement in endocrine signaling and related diseases, particularly in hormone resistance and breast cancer.

### 3.10. RegulomeDB Analysis-Based Identification of High-Confidence Regulatory Variants in ESR2 3’UTR

RegulomeDB v2.0 was used for variant annotation to investigate potential post-transcriptional regulatory elements within the ESR2 3′ UTR. The analysis identified 34 variants in the ESR2 3′ UTR that are predicted to influence gene regulation through altered miRNA binding, mRNA stability, and translation efficiency. Across the ESR2 3′UTR, two variants achieved rank-1 (eQTL + binding), while the rest were classified as rank-2b (binding without eQTL). The common polymorphism rs4986938 (chr14:64233097–64233098) was ranked 1b, score 1.00, with AF = 0.35, indicating strong regulatory evidence and broad population relevance. A second rank-1 site, rs113851861 (chr14:64084854–64084855), was ranked 1f, score 0.223, AF = 0.0141. All other sites were ranked 2b, with scores ranging from approximately 0.94 to 0.01 (e.g., rs989397691 = 0.941; rs57659495 = 0.761; rs778324793 = 0.0127) and were rare to ultra-rare (typically AF < 0.005). After removing duplicates, the landscape is dominated by low-frequency regulatory candidates, with only one high-frequency exception (rs4986938) (Table 5).

### 3.11. Identification of Functional miRNA Target Site Variants in ESR2 3’UTR

The analysis identified highly potential ncSNPs and INDELs in the ESR2 gene, which could influence post-transcriptional regulation through altered miRNA binding. The PolymiRTS algorithm pinpointed key variants such as rs139004885, which disrupts four miRNA binding sites (hsa-miR-1185-5p, hsa-miR-5004-5p, hsa-miR-3679-5p, and hsa-miR-5191), with notable reductions in score (−0.16 to −0.229). Conservation analysis found variants in conserved miRNA regions, including rs184960071 in a highly conserved hsa-miR-4704-3p site (conservation class 7, Δcontext+ = −0.126), rs142219923 in a hsa-miR-490-5p site (class 6, Δcontext+ = −332), and rs201485281 in a hsa-miR-5591-3p site (class 6, Δcontext+ = −135). Importantly, the rs192894852 variant created a new high-affinity site for hsa-miR-4648 (Δcontext+ = −0.472) (Table 6).

## 4. Discussion

The *ESR2* gene encodes ERβ1, a putative tumor suppressor in hormone-dependent malignancies. Although ERβ biology has been studied extensively at the expression level, the functional impact of nsSNP and UTR variants remains underexplored. Accordingly, this study focuses on the mechanistic and translational implications of ESR2 coding and regulatory variation, considering how alterations may affect ERβ1 structural stability, ligand- and DNA-binding capacities, co-regulator recruitment, and post-transcriptional regulation, and situates these potential perturbations within relevant pathway and protein–protein interaction contexts. The study aimed to develop an integrative, multi-tool silico framework that prioritizes *ESR2* variant classes, annotates their biological context, and evaluates their potential clinical relevance through pathway/regulatory analyses, as well as pan-cancer survival modeling. This framework generates testable hypotheses to guide experimental validation and inform biomarker and therapeutic development.

The predictive computational tools I-Mutant and MUpro showed how nsSNPs impact the stability of the ERβ1 protein. Most nsSNPs, especially in the LBD and DBD, significantly reduced protein stability, which is vital for ERβ1’s functional integrity. Notably, mutations such as R454C (rs768924970), C149G (rs1351313879), D154G (rs775445438), and L380P (rs1249242790) exhibited strongly negative ΔΔG values on both platforms, indicating a high chance of impaired receptor function and structural instability. Protein stability is a key factor in receptor activity. Changes in these crucial domains may weaken the receptor’s ability to bind DNA and ligands, disrupting estrogen signaling pathways [53]. Additionally, recent studies have found decreased *ESR2* protein stability in patients with PCOS, linked to variants R454C (rs768924970) and L380P (rs1249242790). This suggests these variants may play a role in hormone-related disorders [36].

HOPE-based structure predictions showed that most ERβ1 protein nsSNPs significantly impact protein shape through changes in hydrophobicity, size, and charge of residues in conserved regions. Cysteine residues (C149G, C152Y, C191Y/S/G/R)-affecting mutations are especially harmful, leading to the loss of aromatic interactions and important disulfide bonds [54]. Proline-glycine substitutions (P156R, G352S, G502R) have been reported and could potentially hinder secondary structure formation and backbone flexibility [55]. Charge-changing substitutions (*R197W/Q*, *E211K*, and *D154N/G*) may affect key electrostatic networks related to DNA recognition, dimerization, and ligand binding [56,57,58]. Residue changes within the hydrophobic core (*W535R*, *S157L*, *V370I*) reduce folding stability [59]. Overall, the predictions suggest multiple structural mechanisms—such as disulfide loss, flexibility reduction, steric packing changes, hydrophobic destabilization, and electrostatic disruption—by which *ESR2* nsSNPs could influence receptor function. 

MutPred2 analysis of *ESR2* coding variants was performed to assess the molecular pathogenicity of the identified nsSNPs. It showed that the involvement of cysteine residues (C191, C169, C152, and C149) in mutations could influence disulfide bond formation. This aligns with the effects of conservative cysteine-to-serine changes in the Ca^2+^ receptor, which disrupts cell surface expression and dimerization. C131 and C129 are required explicitly for receptor-function assembly and intermolecular disulfide bonding [60]. Therefore, *ESR2* activity and stability may also depend on a disulfide-mediated interface. Similarly, cysteine mutations could significantly impact receptor conformation and regulatory signaling.

The current study identified sulfation modifications at Y155 as a common molecular outcome in *ESR2*. Sulfation is considered an important post-translational modification involved in endocrine signaling. This modification can significantly affect hormone activity, clearance, and receptor binding [61]. Such changes are critical for the neuroendocrine system and steroid function. Therefore, disrupting sulfation at Y155 in *ESR2* may reduce receptor signaling and stability. 

CScape and CScape-somatic identified the oncogenic potential of *ESR2* gene nsSNPs in the coding region. A wide array of *ESR2* mutants demonstrated significant high-confidence driver potential, with several mutants (R198P, C191G, C149G, and D154N) achieving scores exceeding 0.90 for oncogenicity. The strong concordance between the predictions of CScape and CScape-somatic underscores the pathogenic potential of these mutations.

Notably, the C191 residue emerged as a recurrently mutated hotspot, and its four distinct substitutions (C191Y/S/G/R) were consistently classified as drivers. This finding aligns with prior MuPred2 modeling data (functional and structural), which underscores the critical role of this region in *ESR2*-mediated oncogenic mechanisms. Tumor-specific associations derived from CBioPortal indicate that various high-confidence variants (R197W, R454C, R207Q, G502R, D326N, R388Q, G352S, and V370I) are enriched in particular cancer subtypes, including lung, colorectal, breast, and renal carcinomas. Furthermore, the presence of exceedingly low allele frequencies in population-level data (AF < 0.00001) confirms the somatic nature of these variants and substantiates their function as significant drivers. Collectively, these findings affirm the pathogenicity of *ESR2* modifications. Additionally, the study emphasizes the importance of integrative computational frameworks in prioritizing variants for functional validation within cancer research.

STRING analysis of ERβ1 protein partners revealed a high-confidence interaction network comprising signaling partners (*SP1*, *CAV1,* and *JUN*), coactivators (*NCOA1-3* and *MED1*), and a corepressor (*NCOR1*). This network strongly highlights ERβ1’s role in cross-pathway signaling and transcription regulation. Notably, *NCOA1-3* and *MED1* formed a tightly connected module for ligand-dependent transcription and exhibited the highest STRING scores.

Prioritized DBD variants (e.g., C149G, D154G/D154N) are positioned to weaken ERβ1–DNA interactions [62], while hinge/NLS changes (R198P/C191) could perturb nuclear import and chromatin residency [63]. LBD/AF-2 substitutions (e.g., L380P) may diminish coactivator recruitment (e.g., *NCOA1*/*SRC* family), attenuating ERβ1-mediated transcription. Such alterations intersect pathways implicated in endocrine biology and resistance (ERα crosstalk, AP-1/SP1/NF-κB interfaces, PI3K–AKT/MAPK signaling) [64], offering tumor-specific hypotheses in breast, ovarian, endometrial, and colorectal settings.

Consistently, our KEGG enrichment highlights estrogen signaling, endocrine resistance, and breast cancer pathways, supporting a model wherein these variants may blunt ERβ1’s tumor-suppressive signaling and bias cells toward ER-dependent growth and therapy resistance.

Integrating AF clarifies biological plausibility: ultra-rare coding variants with strong oncogenicity/stability signatures (e.g., C149G, D154N/G, R198P, C191 substitutions) align with a driver profile, whereas common 3′UTR variants (e.g., rs4986938) may contribute to population-modulated susceptibility via post-transcriptional mechanisms. This dual pattern—rare high-impact coding changes and common regulatory polymorphisms—fits *ESR2*’s context-dependent role across hormone-responsive tissues.

Our cross-tumor pattern—*ESR2*-Altered linked to worse OS in BRCA but better OS in UCEC and OV—is broadly consistent with an expression-focused literature suggesting that ERβ activity tends to be protective, while also emphasizing known context and isoform dependencies. In breast cancer, several cohort analyses report that ERβ (especially ERβ1) expression correlates with more favorable outcomes, supporting a tumor-suppressive role; however, results vary by ERβ isoform, cellular localization, *ERα* status, and endocrine therapy exposure, with some series noting adverse signals in specific tamoxifen-treated subgroups. This heterogeneity makes it biologically plausible that a subset of *ESR2* alterations (e.g., loss-of-function mutations) could be associated with poorer OS, as we observed in BRCA [25,29].

In ovarian cancer, meta-analytic evidence indicates that higher ERβ (and ERα) expression is associated with improved survival. However, estimates can vary depending on antibody specificity—consistent with our finding that the *ESR2*-Altered group has better outcomes. Meanwhile, isoform-specific studies reveal different biology: ERβ1 generally has growth-inhibiting effects, whereas ERβ2/ERβ5 can promote migration and invasion, highlighting why combined “Altered” categories and unstratified IHC might hide contrasting effects [30].

For endometrial cancer, multiple analyses associate hormone-receptor positivity (*ER*/*PR*) with better survival, consistent with our UCEC findings. Notably, studies of *ESR2* polymorphisms (e.g., rs1256049, rs4986938) mainly focus on risk rather than prognosis—some report increased endometrial cancer risk for rs1256049, while rs4986938 shows no overall cancer-risk link—highlighting the importance of distinguishing risk alleles from variants that truly affect prognosis when interpreting survival [65].

Together, previous research supports a beneficial ERβ axis in OV and UCEC, and often in BRCA at the expression level. However, our mutation-level analysis indicates that *ESR2* genetic status may have an adverse association in BRCA but a favorable one in OV/UCEC—an apparent divergence likely explained by differences in variant function, isoform balance, subcellular localization, and endocrine context. Future studies that combine variant-level annotation (deleterious vs. likely benign; coding vs. UTR) with isoform-specific IHC/RNA analysis and multivariable modeling should help reconcile mutation- and expression-based prognostics across different tumor types.

A hydrophobic groove, constituted by conserved hydrophobic residues within the LBD, helix 12, and lysine 366, functions as the estrogen receptor coactivator binding site [10]. This site includes a p160 coactivator LXXLL motif, where proximal basic residues enhance high-affinity binding through electrostatic interactions during transcriptional activation [10]. Driver oncogenic nsSNPs within the AF-2/LBD (G352S, D326N, V370I, R454C, and R388Q) may impair coactivator recruitment and diminish *ESR2*-mediated gene activation. Similarly, driver oncogenic nsSNPs in the DBD, located within the 146-217 region (including C169F/R, R207Q, and R197W), may disrupt interactions with *JUN* and *SP1*, thereby affecting estrogen signaling pathways and stress cross-talk. The context-dependent interactions between *CAV1* and *JUN* may represent a potential perturbation of non-genomic signaling pathways. Overall, these findings indicate that mutations associated with cancer could influence the *ESR2* interactome network, potentially leading to uncontrolled transcription and tumorigenesis.

An integrated analysis of *ESR2* nsSNPs pertaining to Gene Ontology (GO) biological processes and KEGG pathways has yielded significant insights into disease pathogenesis and the biology of estrogen receptors. This notable enrichment in intracellular steroid hormone receptor signaling (GO) and endocrine resistance (KEGG) indicates that nsSNPs predominantly influence the functions of classical nuclear receptors within the ligand-binding domain (LBD) and AF-2 region.

The strong enrichment for mammary gland morphogenesis (GO) and breast cancer pathways (KEGG) further confirmed *ESR2* nsSNPs’ roles in oncogenic transformation and tissue homeostasis. In particular, the Cysteine-substitution mutations (C169F/R and C191R/Y) were significant, as they could destabilize the ZnF motifs in the DBD, leading to altered target gene specificity.

The pathway analysis further revealed *ESR2* signaling and other endocrine systems’ extensive cross-talk through the thyroid hormone signaling pathway (KEGG). It indicates that nsSNPs may disrupt the nuclear receptor’s cross-regulation balance and contribute to the complex etiology of endocrine disorders. The concurrent enrichment of prolactin signaling (KEGG) further substantiates that *ESR2* variants could influence multiple axes of hormonal regulation.

RegulomeDB-based regulatory annotation of *ESR2* 3′UTR SNPs reveals SNP enrichment with potential regulatory effects. The rs4986938 SNP is classified as 1b, indicating high confidence from eQTL data, TF binding, DNase footprinting, and motif disruption. The rs113851861 SNP falls into category 1f, emphasizing its significant regulatory role. Most variants are categorized as 2b, which suggests chromatin accessibility and TF binding without supporting eQTL evidence. These results illustrate a complex regulatory network where *ESR2* expression is influenced in a tissue-specific manner. They also support previous findings linking rs4986938 to hormone-related cancer risk. For example, decreased ERβ1 levels in breast cancer reduce protection, while rs4986938 is associated with increased risk in colorectal cancer under high estradiol levels [22].

Variants in miRNA target sites within 3′UTRs may also significantly influence *ESR1* regulation and the susceptibility to breast cancer [20]. The current analysis provides novel insights into *ESR2*, specifically regarding miRNA-target gene interactions. The polymiRTS algorithm enabled the identification of numerous INDELs and ncSNPs in *ESR2 3′UTR*, which exhibit a high likelihood of affecting regulation at the post-transcriptional level, including disruption of miRNA binding. A notable variant, rs139004885, interfered with four distinct miRNAs (hsa-miR-5004-5p, hsa-miR-1185-5p, hsa-miR-5191, and hsa-miR-3679-5p), demonstrating significantly lower binding scores.

The Conservation analysis identified potentially functional rs184960071 and rs142219923 variants within highly conserved regions (conservation classes 7 and 6) of miRNA binding. Additionally, rs192894852 could potentially create a new high-affinity binding site for hsa-miR-4648, indicating a possible gain-of-function regulatory effect. These results align with extensive genomic studies show that variation in miRNA binding sites can influence regulatory interactions and alter gene expression [21]. Therefore, *ESR2* 3′UTRs’ genetic variability might contribute to disease phenotypes through miRNA-dependent regulation.

The current study provides several therapeutic implications. Patients with disruptive *ESR2* nsSNPs might not respond to SERMs/SERDs, which depend on functional ERβ1. It clarifies tamoxifen and fulvestrant resistance in some ERβ1-positive tumors. *ESR2* mutation status could serve as a potential biomarker for selecting patients for ERβ1-targeting agonists; however, their effectiveness in mutation-positive tumors remains untested.

## 5. Conclusions

This comprehensive in silico analysis identifies high-risk *ESR2* variants that are predicted to affect ERβ1’s structural stability, DNA and ligand binding, co-regulator recruitment, and regulatory interactions. Variants such as C149G, D154G/N, R198P, and C191 substitutions stand out as strong candidates for pathogenic causes, while regulatory 3′UTR polymorphisms, including rs4986938, may influence population-specific susceptibility. Overall, these findings reveal the dual landscape of *ESR2* genetic variation, encompassing both rare driver-like mutations and common functional polymorphisms that could impact hormone-dependent tumor biology.

Importantly, our pan-cancer survival analysis revealed tumor-type-specific prognostic patterns: *ESR2* alterations were linked to worse outcomes in breast cancer but better outcomes in ovarian and endometrial cancers. These findings support the idea that ERβ1 signaling plays a context-dependent role and highlight the potential of *ESR2* variants as biomarkers for prognosis, therapy response, and as targets for selective ERβ-modulating treatments. However, since this study is exploratory and based solely on computational predictions, rigorous validation through functional assays, CRISPR knock-in models, and patient cohorts remain essential before any clinical application.

## Figures and Tables

**Table 1:** PredictSNP prediction of the functional impact of nsSNPs within the *ESR2* gene. Each SNP is identified by its rsID and the corresponding amino acid (AA) change. PredictSNP provides a consensus prediction generated by multiple tools. The MAPP, PhD-SNP, SIFT, and SNAP columns show combined outputs from four individual predictors. The PolyPhen-1 and PolyPhen-2 columns indicate structural and functional impacts based on their predictions.

nsSNP rsID	AA Change	PredictSNP	MAPP, PhD-SNP, SIFT, SNAP	PolyPhan-1 and 2
rs1241458487	P44L	Deleterious	Deleterious	Damaging
rs147382781	R93C	Deleterious	Deleterious	Damaging
rs911726841	P106R	Deleterious	Deleterious	Damaging
rs141516067	S112L	Deleterious	Deleterious	Damaging
rs774742997	A134T	Deleterious	Deleterious	Damaging
rs1351313879	C149G	Deleterious	Deleterious	Damaging
rs1353654623	C152Y	Deleterious	Deleterious	Damaging
rs775445438	D154G	Deleterious	Deleterious	Damaging
rs760612953	D154N	Deleterious	Deleterious	Damaging
rs1472743418	A156T	Deleterious	Deleterious	Damaging
rs1016270637	S157L	Deleterious	Deleterious	Damaging
rs1411758930	H160R	Deleterious	Deleterious	Damaging
rs770224156	Y161C	Deleterious	Deleterious	Damaging
rs1261390478	G162R	Deleterious	Deleterious	Damaging
rs141760704	S165L	Deleterious	Deleterious	Damaging
rs1273276574	C169F	Deleterious	Deleterious	Damaging
rs1029338063	C169R	Deleterious	Deleterious	Damaging
rs1457342604	S176R	Deleterious	Deleterious	Damaging
rs1367633095	N189Y	Deleterious	Deleterious	Damaging
rs766405281	C191G	Deleterious	Deleterious	Damaging
rs766405281	C191R	Deleterious	Deleterious	Damaging
rs766405281	C191S	Deleterious	Deleterious	Damaging
rs1305200621	C191Y	Deleterious	Deleterious	Damaging
rs773394073	I193N	Deleterious	Deleterious	Damaging
rs145278854	D194N	Deleterious	Deleterious	Damaging
rs760053106	R197W	Deleterious	Deleterious	Damaging
rs768839285	R198C	Deleterious	Deleterious	Damaging
rs1489920793	R198P	Deleterious	Deleterious	Damaging
rs748841139	R205Q	Deleterious	Deleterious	Damaging
rs371856990	R207W	Deleterious	Deleterious	Damaging
rs368924653	R207Q	Deleterious	Deleterious	Damaging
rs909370443	K208M	Deleterious	Deleterious	Damaging
rs556956556	E211K	Deleterious	Deleterious	Damaging
rs1410117865	M214V	Deleterious	Deleterious	Damaging
rs1290256152	M214I	Deleterious	Deleterious	Damaging
rs1190163038	R220Q	Deleterious	Deleterious	Damaging
rs1307959271	R227C	Deleterious	Deleterious	Damaging
rs755401425	D236Y	Deleterious	Deleterious	Damaging
rs766843910	E237K	Deleterious	Deleterious	Damaging
rs576722274	K244E	Deleterious	Deleterious	Damaging
rs1220163606	R247K	Deleterious	Deleterious	Damaging
rs543025691	A252T	Deleterious	Deleterious	Damaging
rs1199203068	R256W	Deleterious	Deleterious	Damaging
rs768870975	P277Q	Deleterious	Deleterious	Damaging
rs1436572414	P277S	Deleterious	Deleterious	Damaging
rs1488031774	T290I	Deleterious	Deleterious	Damaging
rs747036560	D303N	Deleterious	Deleterious	Damaging
rs539389612	K304T	Deleterious	Deleterious	Damaging
rs550448628	W312C	Deleterious	Deleterious	Damaging
rs138920605	P317T	Deleterious	Deleterious	Damaging
rs2229618	L322V	Deleterious	Deleterious	Damaging
rs905821436	D326N	Deleterious	Deleterious	Damaging
rs145661652	R329Q	Deleterious	Deleterious	Damaging
rs1276302781	L330H	Deleterious	Deleterious	Damaging
rs149090049	W335R	Deleterious	Deleterious	Damaging
rs755668062	L339P	Deleterious	Deleterious	Damaging
rs755668062	L339Q	Deleterious	Deleterious	Damaging
rs553390407	G342E	Deleterious	Deleterious	Damaging
rs553390407	G342V	Deleterious	Deleterious	Damaging
rs1194417609	W345C	Deleterious	Deleterious	Damaging
rs200264592	R346C	Deleterious	Deleterious	Damaging
rs1339881550	R346H	Deleterious	Deleterious	Damaging
rs368060197	G352S	Deleterious	Deleterious	Damaging
rs1294597204	A357V	Deleterious	Deleterious	Damaging
rs141474553	R364S	Deleterious	Deleterious	Damaging
rs745947456	V370I	Deleterious	Deleterious	Damaging
rs1249242790	L380P	Deleterious	Deleterious	Damaging
rs112017626	L381P	Deleterious	Deleterious	Damaging
rs1010629502	R386G	Deleterious	Deleterious	Damaging
rs764756707	R388Q	Deleterious	Deleterious	Damaging
rs764756707	R388P	Deleterious	Deleterious	Damaging
rs1012693115	Y397H	Deleterious	Deleterious	Damaging
rs1197276001	L398R	Deleterious	Deleterious	Damaging
rs1432744457	M403R	Deleterious	Deleterious	Damaging
rs111471356	L406R	Deleterious	Deleterious	Damaging
rs778031158	N407Y	Deleterious	Deleterious	Damaging
rs78255744	S408F	Deleterious	Deleterious	Damaging
rs2229618	L413V	Deleterious	Deleterious	Damaging
rs1217623435	L426R	Deleterious	Deleterious	Damaging
rs755668062	L430P	Deleterious	Deleterious	Damaging
rs768924970	R454C	Deleterious	Deleterious	Damaging
rs775944471	L455P	Deleterious	Deleterious	Damaging
rs375446581	L462P	Deleterious	Deleterious	Damaging
rs767658683	E474G	Deleterious	Deleterious	Damaging
rs1451169980	V485A	Deleterious	Deleterious	Damaging
rs528840784	R501C	Deleterious	Deleterious	Damaging
rs766524153	G502R	Deleterious	Deleterious	Damaging
rs565210086	P514L	Deleterious	Deleterious	Damaging
rs757686092	S529F	Deleterious	Deleterious	Damaging
rs757686092	S529F	Deleterious	Deleterious	Damaging

**Table 2:** I-Mutant and MUpro analyses of nsSNPs predicting their effects on *ESR2* protein stability. The table lists SNP ID, the associated amino acid (AA) change, and the relevant protein domain (N-terminal, DBD, hinge, or LBD). I-Mutant predictions include stability change (increase/decrease), RI, and ΔΔG (kcal/mol).

SNP ID	AA Change	Protein Domain	I-Mutant	RI	DDG-Free Energy Change Value	MUpro	DDG
rs1241458487	P44L	AF-1	Decrease	3	−0.68	Increase	0.12
rs147382781	R93C	AF-1	Decrease	5	−1.76	Decrease	−0.59
rs911726841	P106R	AF-1	Decrease	4	−0.25	Decrease	−1.13
rs141516067	S112L	AF-1	Increase	0	0.14	Increase	0.16
rs774742997	A134T	AF-1	Decrease	4	−0.18	Decrease	−0.71
rs1351313879	C149G	DBD	Decrease	8	−3.26	Decrease	−1.71
rs1353654623	C152Y	DBD	Decrease	2	0.2	Decrease	−0.74
rs775445438	D154G	DBD	Decrease	4	−2.25	Decrease	−1.88
rs760612953	D154N	DBD	Decrease	3	−0.33	Decrease	−1.17
rs1472743418	A156T	DBD	Decrease	6	−0.5	Decrease	−1.27
rs1016270637	S157L	DBD	Decrease	4	−0.22	Decrease	−0.1
rs1411758930	H160R	DBD	Decrease	7	−0.28	Decrease	−0.82
rs770224156	Y161C	DBD	Decrease	5	−0.14	Decrease	−0.93
rs1261390478	G162R	DBD	Decrease	8	0.07	Decrease	−0.63
rs141760704	S165L	DBD	Increase	0	−0.09	Increase	0.37
rs1273276574	C169F	DBD	Decrease	3	0.02	Decrease	−0.86
rs1029338063	C169R	DBD	Decrease	5	−0.67	Decrease	−1.19
rs1457342604	S176R	DBD	Decrease	3	−1.17	Decrease	−0.78
rs1367633095	N189Y	DBD	Decrease	3	−0.01	Decrease	−0.39
rs766405281	C191G	DBD	Decrease	6	−1.18	Decrease	−1.85
rs766405281	C191R	DBD	Decrease	5	−0.57	Decrease	−1.2
rs766405281	C191S	DBD	Decrease	6	−0.64	Decrease	−1.56
rs1305200621	C191Y	DBD	Decrease	4	−0.01	Decrease	−1.03
rs773394073	I193N	DBD	Decrease	8	−0.65	Decrease	−1.98
rs145278854	D194N	DBD	Decrease	6	−0.45	Decrease	−1.01
rs760053106	R197W	DBD	Decrease	5	0.04	Decrease	−1.1
rs768839285	R198C	DBD	Decrease	4	−0.14	Decrease	−1.03
rs1489920793	R198P	DBD	Decrease	4	−0.96	Decrease	−1.59
rs748841139	R205Q	DBD	Decrease	9	−0.87	Decrease	−0.69
rs371856990	R207W	DBD	Decrease	6	0.02	Decrease	−1.184
rs368924653	R207Q	DBD	Decrease	9	−0.84	Decrease	−1.17
rs909370443	K208M	DBD	Increase	4	0.67	Decrease	−0.12
rs556956556	E211K	DBD	Decrease	9	−1.37	Decrease	−0.67
rs1410117865	M214V	DBD	Decrease	7	−0.62	Decrease	−0.95
rs1290256152	M214I	DBD	Decrease	7	−0.84	Decrease	−0.67
rs1190163038	R220Q	DBD	Decrease	9	−1.51	Decrease	−0.55
rs1307959271	R227C	DBD	Decrease	3	−0.26	Decrease	−0.32
rs755401425	D236Y	DBD	increase	0	−0.31	Decrease	−0.81
rs766843910	E237K	DBD	Decrease	8	−0.93	Decrease	−0.65
rs576722274	K244E	DBD	Decrease	4	0.06	Decrease	−0.14
rs1220163606	R247K	DBD	Decrease	8	−0.99	Decrease	−0.82
rs543025691	A252T	DBD	Decrease	5	−0.22	Decrease	−0.2
rs1307959271	R227C	Hinge	Decrease	3	−0.26	Decrease	−0.32
rs1199203068	R256W	LBD	Decrease	7	−0.47	Decrease	−0.08
rs768870975	P277Q	LBD	Decrease	8	−1.93	Decrease	−0.86
rs1436572414	P277S	LBD	Decrease	8	−1.90	Decrease	−1.08
rs1488031774	T290I	LBD	Increase	6	1.03	Decrease	−0.23
rs747036560	D303N	LBD	Decrease	2	−0.16	Decrease	−1.11
rs539389612	K304T	LBD	Increase	5	−0.01	Decrease	−0.77
rs550448628	W312C	LBD	Increase	2	0.35	Decrease	−0.77
rs138920605	P317T	LBD	Decrease	8	−1.82	Decrease	−1.23
rs2229618	L322V	LBD	Increase	7	0.48	Decrease	−0.99
rs905821436	D326N	LBD	Decrease	2	−0.78	Decrease	−0.87
rs145661652	R329Q	LBD	Decrease	9	−1.31	Decrease	−0.43
rs1276302781	L330H	LBD	Decrease	8	−2.42	Decrease	−2.64
rs149090049	W335R	LBD	Decrease	7	−1.57	Decrease	−1.17
rs755668062	L339P	LBD	Increase	5	0.35	Decrease	−1.96
rs755668062	L339Q	LBD	Decrease	3	−0.37	Decrease	−1.61
rs553390407	G342E	LBD	Increase	3	0.56	Decrease	−0.6
rs553390407	G342V	LBD	Decrease	5	−0.64	Decrease	−0.24
rs1194417609	W345C	LBD	Decrease	5	−0.47	Decrease	−0.85
rs200264592	R346C	LBD	Decrease	6	−1.6	Decrease	−0.78
rs1339881550	R346H	LBD	Decrease	8	−0.89	Decrease	−1.36
rs368060197	G352S	LBD	Decrease	8	−1.25	Decrease	−0.66
rs1294597204	A357V	LBD	Decrease	3	−0.21	Decrease	−0.49
rs141474553	R364S	LBD	Decrease	8	−0.69	Decrease	−1.38
rs745947456	V370I	LBD	Decrease	8	−1.01	Decrease	−0.58
rs1249242790	L380P	LBD	Decrease	8	−3.44	Decrease	−2.26
rs112017626	L381P	LBD	Decrease	8	−2.25	Decrease	−1.99
rs1010629502	R386G	LBD	Decrease	9	−2.46	Decrease	−1.38
rs764756707	R388Q	LBD	Decrease	5	−0.14	Decrease	−0.7
rs764756707	R388P	LBD	Decrease	7	−1.66	Decrease	−1.05
rs1012693115	Y397H	LBD	Decrease	8	−1.27	Decrease	−1.66
rs1197276001	L398R	LBD	Decrease	3	0.12	Decrease	−1.54
rs1432744457	M403R	LBD	Increase	3	0.79	Decrease	−1.81
rs111471356	L406R	LBD	Increase	2	0.45	Decrease	−1.49
rs778031158	N407Y	LBD	Decrease	7	−0.97	Decrease	−0.48
rs2229618	L413V	LBD	Decrease	8	−0.86	Decrease	−0.88
rs1217623435	L426R	LBD	Decrease	3	−0.02	Decrease	−1.44
rs755668062	L430P	LBD	Increase	6	−0.18	Decrease	−1.85
rs768924970	R454C	LBD	Decrease	1	−0.36	Decrease	−0.74
rs775944471	L455P	LBD	Increase	4	−0.45	Decrease	−2.28
rs375446581	L462P	LBD	Increase	2	1	Decrease	−1.9
rs767658683	E474G	LBD	Decrease	6	−0.46	Decrease	−1.37
rs1451169980	V485A	LBD	Decrease	1	−0.69	Decrease	−1.03
rs528840784	R501C	LBD	Decrease	5	−0.75	Decrease	−0.77
rs766524153	G502R	LBD	Decrease	8	−0.81	Decrease	−0.65
rs565210086	P514L	LBD	Decrease	1	−0.5	Decrease	−0.05
rs757686092	S529F	LBD	Increase	7	1.52	Increase	0.33

**Table 3:** MuPred2-based predictions of pathogenicity and associated molecular mechanisms for selected *ESR2* missense nsSNPs. The MuPred2 score (ranging from 0 to 1) indicates the probability of pathogenicity, with higher scores denoting a greater predicted impact. Molecular mechanisms with statistical significance (*p* < 0.05) are presented alongside the corresponding structural alterations.

AA Change	MuPred2 Score	Molecular Mechanisms at *p* < 0.05
C149G	0.868	Gain of Strand Altered Stability
C152Y	0.899	Altered Ordered interface. Gain of Sulfation at Y155
D154G	0.849	Altered Ordered interface. Loss of Sulfation at Y155
D154N	0.723	Loss of Loop Loss of Sulfation at Y155
S157L	0.82	Altered Ordered interface. Loss of Sulfation at Y155
H160R	0.897	Altered Ordered interface. Loss of Sulfation at Y155
Y161C	0.851	Altered Ordered interface. Altered Metal binding
GI62R	0.91	Gain of Helix Altered Ordered interface. Altered Metal binding
C169F	0.919	Altered Metal binding. Altered Disordered interface Altered Transmembrane protein. Gain of Disulfide linkage at C166
C169R	0.936	Altered Metal binding. Altered Disordered interface. Gain of Acetylation at K174 Altered Transmembrane protein. Gain of Disulfide linkage at C166
S176R	0.899	Gain of ADP-ribosylation at S176 Altered Transmembrane protein
N189Y	0.868	Altered Disordered interface. Loss of Disulfide linkage at C191 Altered Ordered interface. Altered Metal binding Altered Transmembrane protein Loss of GPI-anchor amidation at N189
C191G	0.898	Loss of Disulfide linkage at C191 Altered Disordered interface. Loss of helix Altered Transmembrane protein Altered DNA binding. Altered Stability Gain of GPI-anchor amidation at N189
C191R	0.927	Loss of Disulfide linkage at C191 Altered Disordered interface. Altered Transmembrane protein. Altered DNA binding. Loss of GPI-anchor amidation at N189
C191S	0.852	Loss of Disulfide linkage at C191 Altered Disordered interface. Altered Transmembrane protein. Altered DNA binding. Gain of N-linked glycosylation at N189 Gain of GPI-anchor amidation at N189
C191Y	0.892	Altered Disordered interface. Loss of Disulfide linkage at C191 Altered Transmembrane protein. Loss of Helix Altered DNA binding. Loss of GPI-anchor amidation at N189
R197W	0.852	Altered DNA binding. Loss of Helix Altered Disordered interface. Altered Transmembrane protein
R198C	0.867	Altered Disordered interface. Loss of Helix Altered DNA binding. Altered Metal binding
R198P	0.95	Loss of Helix Altered Disordered interface Altered DNA binding
R205Q	0.727	Altered Disordered interface.
L330H	0.847	Loss of Helix Altered Stability
W335R	0.885	Gain of Helix
N407Y	0.809	Altered Ordered interface Loss of GPI-anchor amidation at N407
R454C	0.635	Gain of Pyrrolidone carboxylic acid at Q449
D194N	0.841	Altered Disordered interface Altered DNA binding Gain of Disulfide linkage at C191, Altered Transmembrane protein Gain of GPI-anchor amidation at N189
R207Q	0.609	Altered Disordered interface
D303N	0.742	Loss of Ubiquitylation at K300

**Table 4:** CScape-somatic (v1.0) and CScape (v2.0) algorithms-based prediction of oncogenic impact of somatic missense nsSNPs.

			CScape		CScape-Somatic				
Variant ID	AA Changes	Input, Assembly	Coding Scores	Message	Coding Score	Message	GenomeAD AF	CBioPortal AF	Cancer
rs1351313879	C149G	14,64746789,A,C	0.929542	Oncogenic (high conf.)	0.620587	Driver	0.00001487		
rs1353654623	C152Y	14,64746779,C,T	0.925819	Oncogenic (high conf.)	0.356178	Passenger	6.195 × 10^−7^		
rs775445438	D154G	14,64746773,T,C	0.92916	Oncogenic (high conf.)	0.724278	Driver	0.000008674		
rs760612953	D154N	14,64746774,C,T	0.962646	Oncogenic (high conf.)	0.827846	Driver	0.000001859		
rs1016270637	S157L	14,64746764,G,A	0.963144	Oncogenic (high conf.)	0.815778	Driver	0.000002478		
rs1411758930	H160R	14,64746755,T,C	0.939813	Oncogenic (high conf.)	0.604905	Driver	0.000004956		
rs770224156	Y161C	14,64746752,T,C	0.926715	Oncogenic (high conf.)	0.62281	Driver	0.000004956		
rs1261390478	G162R	14,64746750,C,T	0.932607	Oncogenic (high conf.)	0.364078	Passenger	0.000002478		
rs1273276574	C169F	14,64746728,C,A	0.95865	Oncogenic (high conf.)	0.619302	Driver	0.000002478		
rs1029338063	C169R	14,64746729,A,G	0.919354	Oncogenic (high conf.)	0.630163	Driver	0.000001240		
rs1457342604	S176R	14,64746708,T,G	0.942129	Oncogenic (high conf.)	0.521914	Driver	0.000001240		
rs1367633095	N189Y	14,64735600,T,A	0.906308	Oncogenic (high conf.)	0.301533	passenger	0.000001240		
rs766405281	C191G	14,64735594,A,C	0.965581	Oncogenic (high conf.)	0.765388	Driver	6.199 × 10^−7^		
rs766405281	C191R	14,64735594,A,G	0.913891	Oncogenic (high conf.)	0.548061	Driver	6.199 × 10^−7^		
rs766405281	C191S	14,64735594,A,T	0.910363	Oncogenic (high conf.)	0.473874	Passenger	6.199 × 10^−7^		
rs369253565	C191Y	14,64735593,C,T	0.930841	Oncogenic (high conf.)	0.410529	Passenger	0.000008056		
rs760053106	R197W	14,64735576,G,A	0.756852	Oncogenic	0.685594	Driver	0.000009295	0.33/0.37	Colon Adenocarcinoma/Papillary/Stomach Adenocarcinoma
rs768839285	R198C	14,64735573,G,A	0.923613	Oncogenic (high conf.)	0.744457	Driver	6.196 × 10^−7^		
rs1489920793	R198P	14,64735572,C,G	0.974356	Oncogenic (high conf.)	0.847731	Driver	0.000008675		
rs748841139	R205Q	14,64735551,C,T	0.973985	Oncogenic (high conf.)	0.746492	Driver	0.00004091		
rs368924653	R207Q	14,64735545,G,T	0.643141	oncogenic	0.7	Driver	0.000008305	0.29	Stomach Adenocarcinoma
rs766843910	E237K	14,64727410,T,C	0.595541	oncogenic	0.534785	Driver	0.000003916		
rs747036560	D303N	14,64727212,G.T	0.745124	oncogenic	0.45	Passenger	0.00001248	0.23	Chromophobe Renal Cell Carcinoma
rs138920605	P317T	14,64727170,G,T	0.950698	Oncogenic (high conf.)	0.155234	Passenger	0.000005579		
rs905821436	D326N	14,64724059,C,T	0.59872	Oncogenic	0.735066	Driver	—	0.23	Breast Invasive Ductal Carcinoma
rs1276302781	L330H	14,64724046,A,T	0.946816	Oncogenic (high conf.)	0.41106	Passenger	0.000002479		
rs149090049	W335R	14,64724032,A,G	0.917757	Oncogenic (high conf.)	0.534854	Driver	6.195 × 10^−7^		
rs553390407	G342V	14,64724010,C,A	0.941398	Oncogenic (high conf.)	0.355589	Passenger	0.00004832		
rs368060197	G352S	14,64723981,C,T	0.552654	oncogenic	0.667455	Driver	0.000001869	0.38	Uterine Endometrioid Carcinoma
rs141474553	R364S	14,64716397,C,A	0.909262	Oncogenic (high conf.)	0.220505	Passenger	0.00001054		
rs745947456	V370I	14,64716381,C,T	0.628998	oncogenic	0.854778	Driver	0.00004276	0.04	Renal Clear Cell Carcinoma
rs764756707	R388Q	14,64716326,A,T	0.536837	oncogenic	0.857166	Driver	—	0.13	Lung Squamous Cell Carcinoma
rs778031158	N407Y	14,64716270,T,A	0.926747	Oncogenic (high conf.)	0.263806	Passenger	0.000006815		
rs768924970	R454C	14,64701734,G,A	0.921404	Oncogenic (high conf.)	0.676257	Driver	0.00001549		
rs766524153	G502R	14,64699944,T,C	0.604477	oncogenic	0.577366	Driver	0.00001487	0.34	Rectal Adenocarcinoma

AF: global minor allele frequency from gnomAD (GRCh38); rsIDs mapped through Ensembl/dbSNP and spot-checked in UCSC Genome Browser (hg38). Ultra-rare is defined as AF < 1 × 10^−5^. ‘—’ indicates not observed or insufficient counts. AFs are provided to contextualize functional predictions and do not imply causal attribution.

**Table 5:** Annotation and prediction of regulatory potential for ESR2 3′ UTR variants based on RegulomeDB. Data include chromosomal position, dbSNP identifier, functional rank, and regulatory score (0 to 1).

Chromosome Location	dbSNP IDs	Rank	Score	AF
chr14:64233097..64233098	rs4986938	1b	1	0.35
chr14:64233107..64233108	rs989397691	2b	0.94115	0.000001247
chr14:64234904..64234905	rs1023533876	2b	0.88523	0.000003173
chr14:64233105..64233106	rs772987842	2b	0.88523	0.000002502
chr14:64234928..64234929	rs200359377	2b	0.8662	0.002494
chr14:64233116..64233117	rs1418402420	2b	0.84289	6.220 × 10^−7^
chr14:64234924..64234925	rs778636549	2b	0.84289	0.0003263
chr14:64233128..64233129	rs374618716	2b	0.8368	0.000004343
chr14:64234946..64234947	rs1221900232	2b	0.8288	0.000001865
chr14:64233121..64233122	rs1300652813	2b	0.82541	0.000001242
chr14:64234923..64234924	rs370541364	2b	0.79882	0.00001939
chr14:64233101..64233102	rs1306618171	2b	0.7614	6.261 × 10^−7^
chr14:64234898..64234899	rs767381562	2b	0.7614	0.00004845
chr14:64234925..64234926	rs57659495	2b	0.7614	0.003142
chr14:64234927..64234928	rs757989022	2b	0.7614	0.000001250
chr14:64233127..64233128	rs1414551434	2b	0.76026	0.000003102
chr14:64233127..64233128	rs1414551434	2b	0.76026	0.000003102
chr14:64234934..64234935	rs1487252486	2b	0.7516	0.000001248
chr14:64234945..64234946	rs986361217	2b	0.7516	0.000004980
chr14:64233122..64233123	rs915165791	2b	0.75124	6.209 × 10^−7^
chr14:64234936..64234937	rs1477356325	2b	0.74417	0.000001247
chr14:64234933..64234934	rs768095347	2b	0.73193	0.000001248
chr14:64234929..64234930	rs563871991	2b	0.69075	0.000004375
chr14:64234905..64234906	rs909213490	2b	0.67136	0.000003173
chr14:64234940..64234941	rs1444732684	2b	0.61749	0.000002493
chr14:64234893..64234894	rs969459185	2b	0.61652	0.000001917
chr14:64234893..64234894	rs969459185	2b	0.61652	0.000001917
chr14:64234892..64234893	rs756997470	2b	0.51348	0.000004471
chr14:64233123..64233124	rs762341251	2b	0.49614	0.00007760
chr14:64234891..64234892	rs1320955636	2b	0.40513	0.000001279
chr14:64234937..64234938	rs1369678323	2b	0.35835	0.000002494
chr14:64233098..64233099	rs769561814	2b	0.34744	0.000001879
chr14:64084854..64084855	rs113851861	1f	0.22271	0.01410
chr14:64234943..64234944	rs778324793	2b	0.01267	0.000003736

**Table 6:** PolymiRTS analysis-based identification of polymorphic miRNA target sites in ESR2. The table lists genomic coordinates (GRCh38), dbSNP IDs, variant types, ancestral/alternate alleles, affected miRNAs, conservation classes (1–7, with higher numbers indicating higher conservation), miRNA binding sequences (lowercase indicates the seed region), functional classes (D = disruptive, C = constructive, N = neutral, O = other), experimental support (Y/N), and changes in context+ score (negative values suggest reduced binding affinity). Variants highlighted demonstrate strong predicted effects on miRNA regulation.

Location	dbSNP ID	Variant	Wobble	Ancestral	Allele	miR ID	Conservation	miRSite	Function	Exp	Context+
		Type	Base Pair	Allele					Class	Support	Score Change
64693801	rs139004885	SNP	Y	A	A	hsa-miR-1185-5p	3	atgccTATCCTCt	D	N	−0.16
						hsa-miR-3679-5p	3	atgccTATCCTCt	D	N	−0.188
						hsa-miR-5191	2	atgCCTATCCtct	D	N	−0.229
					G	hsa-miR-5004-5p	3	atgccTGTCCTCt	C	N	−0.211
64693871	rs1255998	SNP	N	C	C	hsa-miR-1207-5p	1	gctgtgCCTGCCA	N	N	−0.163
						hsa-miR-4269	1	gctGTGCCTGcca	N	N	−0.181
						hsa-miR-4736	1	gctgtgCCTGCCA	N	N	−0.176
						hsa-miR-4763-3p	1	gctgtgCCTGCCA	N	N	−0.159
						hsa-miR-5582-5p	1	gcTGTGCCTgcca	N	N	−0.093
						hsa-miR-6715b-5p	1	gctGTGCCTGcca	N	N	−0.181
						hsa-miR-7150	1	gctgtgCCTGCCA	N	N	−0.241
					G	hsa-miR-449b-3p	1	gcTGTGGCTgcca	C	N	−0.125
						hsa-miR-4691-3p	1	gctGTGGCTGcca	C	N	−0.211
						hsa-miR-885-3p	1	gctgtgGCTGCCA	C	N	−0.196
64693912	rs58262369	SNP	Y	G	G	hsa-miR-3191-5p	4	ttggCAGAGAAgg	D	N	−0.047
						hsa-miR-6728-3p	5	ttgGCAGAGAagg	D	N	−0.11
						hsa-miR-942-5p	4	ttggcAGAGAAGg	D	N	−0.011
					A	hsa-miR-1248	3	ttggcaAAGAAGG	C	N	−0.12
						hsa-miR-6844	4	ttggCAAAGAAgg	C	N	0.005
64694082	rs8018687	SNP	Y	A	A	hsa-miR-5004-3p	2	cAATCCAAcaatt	D	N	−0.109
64694146	rs114629727	SNP	Y	A	A	hsa-miR-130b-5p	1	aaaGAAAGAGtta	N	N	−0.207
						hsa-miR-618	1	aaagaaAGAGTTA	N	N	−0.112
					G	hsa-miR-1237-3p	1	aaAGAAGGAgtta	C	N	−0.106
						hsa-miR-1248	1	aAAGAAGGAgtta	C	N	−0.303
						hsa-miR-4764-3p	1	aaagaaGGAGTTA	C	N	−0.255
						hsa-miR-6868-3p	1	aaAGAAGGAgtta	C	N	−0.205
64694158	rs28440970	SNP	Y	A	A	hsa-miR-4282	2	aaaaatAATTTTA	D	N	0.071
						hsa-miR-944	1	aaaAATAATTtta	N	N	−0.067
					G	hsa-miR-1250-3p	1	aAAAATGAtttta	C	N	0.023
						hsa-miR-153-5p	1	AAAAATGAtttta	C	N	−0.058
						hsa-miR-4668-3p	2	aaaaatGATTTTA	C	N	−0.008
64694170	rs142219923	SNP	N	C	C	hsa-miR-1246	5	gtAATCCATgaaa	D	N	−0.157
						hsa-miR-490-5p	6	gtaATCCATGAaa	D	N	−0.332
64694195	rs928554	SNP	Y	A	G	hsa-miR-4738-3p	1	acttCAGTTTCcc	C	N	−0.09
					A	hsa-miR-3616-5p	1	ACTTCAAtttccc	N	N	−0.132
						hsa-miR-573	1	ACTTCAAtttccc	N	N	−0.131
64694215	rs201485281	SNP	N	T	T	hsa-miR-5591-3p	6	aaatctTGGGTAA	D	N	−0.135
					C	hsa-miR-4740-3p	2	aaaTCTCGGGtaa	C	N	−0.317
64694234	rs202224619	SNP	N	G	G	hsa-miR-4717-5p	2	aactcgGTGGCCT	D	N	−0.21
64694235	rs74604027	SNP	Y	G	A	hsa-miR-181a-2-3p	2	caacTCAGTGGcc	C	N	−0.183
						hsa-miR-219a-1-3p	3	CAACTCAgtggcc	C	N	−0.145
						hsa-miR-4252	2	caactCAGTGGCc	C	N	−0.219
						hsa-miR-4682	3	cAACTCAGtggcc	C	N	−0.11
						hsa-miR-571	3	CAACTCAgtggcc	C	N	−0.082
64694264	rs141329819	SNP	N	C	C	hsa-miR-181a-3p	1	aagcaaCGATGGA	N	N	−0.173
					T	hsa-miR-4317	1	aaGCAATGAtgga	C	N	−0.086
						hsa-miR-6884-3p	1	aagcaaTGATGGA	C	N	−0.069
64694308	rs146994281	SNP	N	C	C	hsa-miR-3659	1	AACACTCAcctca	N	N	−0.278
						hsa-miR-4658	1	aACACTCAcctca	N	N	−0.16
						hsa-miR-574-5p	1	aACACTCAcctca	N	N	−0.091
						hsa-miR-6134	2	aacactCACCTCA	D	N	−0.119
						hsa-miR-6790-5p	1	aACACTCAcctca	N	N	−0.17
						hsa-miR-7854-3p	2	aacacTCACCTCA	D	N	−0.336
					A	hsa-miR-3591-5p	1	aACACTAAcctca	C	N	−0.119
						hsa-miR-377-5p	2	aacactAACCTCA	C	N	−0.101
						hsa-miR-4255	2	AACACTAacctca	C	N	−0.057
						hsa-miR-6086	2	aacactAACCTCA	C	N	−0.098
						hsa-miR-655-5p	2	aacacTAACCTCA	C	N	−0.316
64694323	rs138379290	SNP	Y	G	G	hsa-miR-1245b-5p	4	agaaAAGGCCTct	D	N	−0.233
						hsa-miR-3142	4	agaaAAGGCCTct	D	N	−0.233
						hsa-miR-627-3p	4	AGAAAAGgcctct	D	N	0.062
					A	hsa-miR-3119	3	agaAAAAGCCtct	C	N	−0.103
						hsa-miR-3188	4	agaaaAAGCCTCt	C	N	−0.212
64694347	rs192894852	SNP	Y	G	G	hsa-miR-4708-5p	1	tgtcccGCATCTC	N	N	−0.124
					A	hsa-miR-1233-5p	1	tgTCCCACAtctc	C	N	−0.153
						hsa-miR-1302	1	TGTCCCAcatctc	C	N	−0.221
						hsa-miR-299-3p	1	tgtCCCACATctc	C	N	−0.223
						hsa-miR-4298	1	TGTCCCAcatctc	C	N	−0.221
						hsa-miR-4648	1	tGTCCCACAtctc	C	N	−0.472
						hsa-miR-4654	1	tgTCCCACAtctc	C	N	−0.172
						hsa-miR-4769-5p	1	tgTCCCACAtctc	C	N	−0.178
						hsa-miR-576-3p	1	tgtccCACATCTc	C	N	−0.103
						hsa-miR-6778-5p	1	tgTCCCACAtctc	C	N	−0.156
64694416	rs35874406	INDEL	N	-	A	hsa-miR-129-5p	1	CAAAAAAttgacat	O	N	0.043
64694496	rs184960071	SNP	N	G	G	hsa-miR-6794-3p	4	ctttGAGTGAAag	D	N	−0.121
						hsa-miR-888-5p	3	cTTTGAGTgaaag	D	N	−0.088
					C	hsa-miR-30a-3p	4	ctttgACTGAAAg	C	N	−0.029
						hsa-miR-30d-3p	4	ctttgACTGAAAg	C	N	−0.029
						hsa-miR-30e-3p	4	ctttgACTGAAAg	C	N	−0.029
						hsa-miR-4704-3p	7	cttTGACTGAaag	C	N	−0.126
64694521	rs1152581	SNP	N	G	G	hsa-miR-1323	3	aaaagAGTTTTGg	D	N	−0.054
						hsa-miR-548o-3p	3	aaaagAGTTTTGg	D	N	−0.082
					T	hsa-miR-548c-3p	2	aaaaGATTTTTgg	C	N	−0.027

## Data Availability

Data is contained within the article and Supplementary Materials.

## Supplementary Materials

The following supporting information can be downloaded at: https://www.mdpi.com/article/10.3390/genes16101144/s1, Table S1: Structural and Functional Impact of ESR2 nsSNPs on ER beta protein using HOPE project analysis.; Table S2: A category scheme for classifying genetic variants using Regulome DB based on their likelihood of affecting gene regulation and binding to transcription factors (TFs).; Table S3: Cross-cohort overall survival by *ESR2* alteration status in BRCA, UCEC, and OV.
