# Supplementary material for: In Silico Characterization of Pathogenic ESR2 Coding and UTR Variants as Oncogenic Potential Biomarkers in Hormone-Dependent Cancers
Source: Genes (Basel). 2025 Sep 26;16(10):1144. doi: 10.3390/genes16101144 (PMC12562404; doi:10.3390/genes16101144)
Supplement: Supplementary file 1 [file genes-16-01144-s001.zip › genes-3844244-supplementary.pdf]

Table S1 : **Structural and Functional Impact of ESR2 nsSNPs on ER beta protein using HOPE project analysis.** This table summarizes the predicted structural and functional consequences of nsSNPs in the ESR2 3D structure, as analyzed using the HOPE (Have (y)Our Protein Explained) computational tool. Each entry includes: the dbSNP reference identifier (SNP ID); the specific amino acid substitution (AA Change); whether the mutation introduces a smaller, larger, or similarly sized residue (Size Change); alterations in electrostatic properties (Charge Change); shifts in hydrophobicity (Hydrophobicity Change); the predicted structural consequences (Structural Impact); and the evolutionary conservation status of the affected residue (Conservation). The Structural Impact column details specific molecular disruptions, including loss of critical interactions (e.g., disulfide bonds, ionic interactions, or hydrogen bonding), steric clashes, and potential folding defects. Conservation status indicates either the percentage of sequence conservation across species or known pathogenic associations, with 100% conservation suggesting functionally critical residues. Mutations flagged as pathogenic or located in highly conserved regions may have particularly severe consequences for ESR2 structure and function.

SNP ID	AA Change	Size Change	Charge Change	Hydrophobicity Change	Structural Impact	Conservation
RS13513879	C149G	Smaller (C → G)	Neutral (no change)	Less hydrophobic (C → G)	Loss of interactions; potential folding disruption	100% conserved
RS1353654623	C152Y	Larger (C → Y)	Neutral (no change)	More hydrophobic (C → Y)	Steric clashes; loss of hydrophobic interactions	100% conserved
RS760612953	D154N	Similar size	Loss of negative charge (D → N)	Slightly more hydrophobic (D → N)	Loss of ionic interactions	100% conserved
RS775445438	D154G	Smaller (D → G)	Loss of negative charge (D → G)	Less hydrophobic (D → G)	Loss of ionic interactions; potential folding disruption	100% conserved
RS911726841	P156R	Larger (P → R)	Introduces positive charge (P → R)	More hydrophobic (P → R)	May cause steric clashes ("bumps"); disrupts proline rigidity (affects backbone folding)	100% conserved
RS1016270637	S157L	Larger (S → L)	Neutral (no change)	More hydrophobic (S → L)	Disrupts hydrogen bonds; folding issues	100% conserved
RS1411758930	H160R	Larger (H → R)	Retains positive charge	Similar hydrophobicity	Steric clashes; potential repulsion with other residues	100% conserved

RS770224156	Y161C	Smaller (Y → C)	Neutral (no change)	More hydrophobic (Y → C)	Loss of aromatic interactions; folding disruption	100% conserved
RS1261390478	G162R	Larger (G → R)	Introduces positive charge (G → R)	More hydrophobic (G → R)	Disrupts backbone flexibility (glycine is critical)	100% conserved
RS1029338063	C169R	Larger (C → R)	Introduces positive charge (C → R)	More hydrophobic (C → R)	Steric clashes; repulsion with nearby residues	100% conserved
RS1273276574	C169F	Larger (C → F)	Neutral (no change)	More hydrophobic (C → F)	Steric clashes; loss of disulfide bonds	100% conserved
RS1457342604	S176R	Larger (S → R)	Introduces positive charge (S → R)	More hydrophobic (S → R)	Steric clashes; disrupts hydrogen bonds	100% conserved
RS1367633095	N179Y	Larger (N → Y)	Neutral (no change)	More hydrophobic (N → Y)	Steric clashes; loss of hydrogen bonds	100% conserved
RS1305200621	C191Y	Larger (C → Y)	Neutral (no change)	More hydrophobic (C → Y)	Steric clashes; loss of disulfide bonds	100% conserved
RS766405281	C191S	Smaller (C → S)	Neutral (no change)	Less hydrophobic (C → S)	Loss of disulfide bonds	100% conserved
RS766405281	C191R	Larger (C → R)	Introduces positive charge (C → R)	More hydrophobic (C → R)	Steric clashes; repulsion with nearby residues	100% conserved
RS766405281	C191G	Smaller (C → G)	Neutral (no change)	Less hydrophobic (C → G)	Loss of disulfide bonds; folding disruption	100% conserved
rs145278854	D194N	Similar size	Loss of negative charge (D → N)	Slightly more hydrophobic (D → N)	May lose interactions	Conserved & Pathogenic

RS760053106	R197W	Larger (R → W)	Loss of positive charge (R → W)	More hydrophobic (R → W)	Loss of ionic interactions; folding disruption	100% conserved
RS1489920793	R198P	Smaller (R → P)	Loss of positive charge (R → P)	More hydrophobic (R → P)	Loss of ionic interactions; disrupts backbone flexibility	100% conserved
RS768839285	R198C	Smaller (R → C)	Loss of positive charge (R → C)	More hydrophobic (R → C)	Loss of ionic interactions; folding disruption	100% conserved
RS748841139	R205Q	Smaller (R → Q)	Loss of positive charge (R → Q)	Similar hydrophobicity	Loss of ionic interactions	100% conserved
RS371856990	R207W	Larger (R → W)	Loss of positive charge (R → W)	More hydrophobic (R → W)	Loss of ionic interactions; folding disruption	Not conserved
rs368924653	R207Q	Smaller (R → Q)	Loss of positive charge (R → Q)	Similar hydrophobicity	May lose interactions	Conserved & Pathogenic
rs556956556	E211K	Larger (E → K)	Charge reversal (negative → positive)	Similar hydrophobicity	May cause repulsion with other residues/ligands	Very conserved
rs1190163038	R220Q	Smaller (R → Q)	Loss of positive charge (R → Q)	Similar hydrophobicity	May lose interactions	Very conserved
rs1307959271	R227C	Smaller (R → C)	Loss of positive charge (R → C)	More hydrophobic (R → C)	Loss of external interactions	Very conserved
RS1307959271	R227C	Smaller (R → C)	Loss of positive charge (R → C)	More hydrophobic (R → C)	Loss of ionic interactions; folding disruption	Very conserved
rs766843910	E237K	Larger (E → K)	Charge reversal (negative → positive)	Similar hydrophobicity	May cause repulsion or steric clashes ("bumps")	Pathogenic & conserved

RS766843910	E237K	Larger (E → K)	Charge reversal (negative → positive)	Similar hydrophobicity	Repulsion with nearby residues; steric clashes	Pathogenic & conserved
rs576722274	K244E	Smaller (K → E)	Charge reversal (positive → negative)	Similar hydrophobicity	May cause repulsion with other residues/ligands	Very conserved
rs747036560	D303N	Similar size	Loss of negative charge (D → N)	Slightly more hydrophobic (D → N)	Buried charge loss may destabilize core	Pathogenic
RS747036560	D303N	Similar size	Loss of negative charge (D → N)	Slightly more hydrophobic (D → N)	Loss of ionic interactions	Pathogenic
rs905821436	D326N	Larger (D → N)	Loss of negative charge (D → N)	More hydrophobic (D → N)	Buried mutant may not fit; loss of hydrophobic interactions	Very conserved
RS905821436	D326N	Larger (D → N)	Loss of negative charge (D → N)	More hydrophobic (D → N)	Buried mutant may not fit; loss of hydrophobic interactions	Very conserved
rs145661652	R329Q	Smaller (R → Q)	Loss of positive charge (R → Q)	Similar hydrophobicity	Buried mutation creates empty space in core	Not conserved
RS145661652	R329Q	Smaller (R → Q)	Loss of positive charge (R → Q)	Similar hydrophobicity	Loss of ionic interactions; empty space in core	Not conserved
rs368060197	G352S	Larger (G → S)	Neutral (no change)	More hydrophobic (G → S)	Disrupts backbone flexibility; may distort local structure	Very conserved & Pathogenic
RS368060197	G352S	Larger (G → S)	Neutral (no change)	More hydrophobic (G → S)	Disrupts backbone flexibility (glycine critical)	Very conserved & pathogenic
rs745947456	V370I	Larger (V → I)	Neutral (no change)	Similar hydrophobicity	Buried mutant may not fit	Very conserved & Pathogenic

RS745947456	V370I	Larger (V → I)	Neutral (no change)	Similar hydrophobicity	Buried mutant may not fit	Very conserved & pathogenic
rs764756707	R388Q	Smaller (R → Q)	Loss of positive charge (R → Q)	Similar hydrophobicity	Loss of external interactions	Very conserved & Pathogenic
RS764756707	R388Q	Smaller (R → Q)	Loss of positive charge (R → Q)	Similar hydrophobicity	Loss of ionic interactions	Very conserved & pathogenic
RS778031158	N407Y	Larger (N → Y)	Neutral (no change)	More hydrophobic (N → Y)	Steric clashes; loss of hydrogen bonds	100% conserved
RS768924970	R444C	Smaller (R → C)	Loss of positive charge (R → C)	More hydrophobic (R → C)	Loss of ionic interactions; folding disruption	100% conserved
rs766524153	G502R	Larger (G → R)	Introduces positive charge (G → R)	More hydrophobic (G → R)	Disrupts backbone flexibility; may cause steric clashes/repulsion	Conserved & Pathogenic
RS766524153	G502R	Larger (G → R)	Introduces positive charge (G → R)	More hydrophobic (G → R)	Disrupts backbone flexibility (glycine critical); steric clashes	Conserved & pathogenic
RS138920605	P517T	Smaller (P → T)	Neutral (no change)	Less hydrophobic (P → T)	Loss of proline rigidity; folding disruption	100% conserved
RS1276302781	L530H	Larger (L → H)	Introduces positive charge (L → H)	Less hydrophobic (L → H)	Steric clashes; repulsion with nearby residues	100% conserved
RS149090049	W535R	Smaller (W → R)	Introduces positive charge (W → R)	Less hydrophobic (W → R)	Loss of aromatic interactions; repulsion	100% conserved
RS553390407	G542V	Larger (G → V)	Neutral (no change)	More hydrophobic (G → V)	Disrupts backbone flexibility (glycine critical)	100% conserved
RS141474553	R554S	Smaller (R → S)	Loss of positive charge (R → S)	Less hydrophobic (R → S)	Loss of ionic interactions	100% conserved

Table S2. RegulomeDB category scheme and functional interpretation. Lower category scores indicate increasing evidence that a variant is located in a functional region. Category 1 variants have equivalents in other categories with the additional requirement of eQTL information.

Category	Category scheme	Description
**1a**	eQTL + TF binding + matched TF motif + matched DNase footprint + DNase peak	Likely to affect binding and linked to expression of a gene target
**1b**	eQTL + TF binding + any motif + DNase footprint + DNase peak	Likely to affect binding
**1c**	eQTL + TF binding + matched TF motif + DNase peak	Likely to affect binding
**1d**	eQTL + TF binding + any motif + DNase peak	Likely to affect binding
**1e**	eQTL + TF binding + matched TF motif	Likely to affect binding
**1f**	eQTL + TF binding/DNase peak	Likely to affect binding
**2a**	TF binding + matched TF motif + matched DNase footprint + DNase peak	Likely to affect binding
**2b**	TF binding + any motif + DNase footprint + DNase peak	Likely to affect binding
**2c**	TF binding + matched TF motif + DNase peak	Likely to affect binding
**3a**	TF binding + any motif + DNase peak	Less likely to affect binding
**3b**	TF binding + matched TF motif	Less likely to affect binding
**4**	TF binding + DNase peak	Minimal binding evidence
**5**	TF binding or DNase peak	Minimal binding evidence
**6**	Motif hit	Minimal binding evidence

Abbreviations: TF, transcription factor; eQTL, expression quantitative trait locus; DNase, deoxyribonuclease.

Table S3. Cross-cohort overall survival by *ESR2* alteration status in BRCA, UCEC, and OV.

Tumor	N (OS)	Altered n (events)	Unaltered n (events)	Median OS Altered (mo; 95% CI)	Median OS Unaltered (mo; 95% CI)	HR (Altered vs Unaltered)	Log-rank p	FDR q
BRCA	6235	57 (24)	6178 (1683)	75.23 (51.20–NA)	152.93 (146.39–164.57)	2.251 (1.235–4.100)	4.98e-05	0.000125
UCEC	1689	43 (3)	1646 (328)	nan	nan	0.239 (0.135–0.424)	0.00717	0.0143
OV	3238	57 (10)	3181 (1245)	106.88 (95.07–NA)	58.05 (55.43–62.12)	0.293 (0.208–0.413)	3.841e-05	0.0001537

Notes: Times are in months. Altered = any reported *ESR2* mutation or nsSNP; Unaltered = no reported *ESR2* alteration. Kaplan–Meier comparisons used the log-rank test; false discovery rate (FDR) control provided q-values when available. Hazard ratios (HRs) are from univariate Cox models (Altered vs Unaltered). Medians reported as “NA” when not reached.

Abbreviations: BRCA, breast carcinoma; UCEC, uterine corpus endometrial carcinoma; OV, ovarian cancer; OS, overall survival; PFS, progression-free survival; DFS, disease-free survival; RFS, relapse-free survival; DSS, disease-specific survival; HR, hazard ratio; FDR, false discovery rate; NA, not reached.
